# Predicting the Enthalpy and Gibbs Energy of Sublimation by QSPR Modeling

**DOI:** 10.1038/s41598-018-28105-6

**Published:** 2018-06-27

**Authors:** Nastaran Meftahi, Michael L. Walker, Marta Enciso, Brian J. Smith

**Affiliations:** La Trobe Institute for Molecular Science, La Trobe University, Melbourne, Victoria 3086 Australia

## Abstract

The enthalpy and Gibbs energy of sublimation are predicted using quantitative structure property relationship (QSPR) models. In this study, we compare several approaches previously reported in the literature for predicting the enthalpy of sublimation. These models, which were reproduced successfully, exhibit high correlation coefficients, in the range 0.82 to 0.97. There are significantly fewer examples of QSPR models currently described in the literature that predict the Gibbs energy of sublimation; here we describe several models that build upon the previous models for predicting the enthalpy of sublimation. The most robust and predictive model constructed using multiple linear regression, with the fewest number of descriptors for estimating this property, was obtained with an R^2^ of the training set of 0.71, an R^2^ of the test set of 0.62, and a standard deviation of 9.1 kJ mol^−1^. This model could be improved by training using a neural network, yielding an R^2^ of the training and test sets of 0.80 and 0.63, respectively, and a standard deviation of 8.9 kJ mol^−1^.

## Introduction

Determination of gas-phase physical properties of small molecules using state-of-the–art computational methods, using either quantum mechanical or density functional methods, has become usual. For compounds that one would expect to find in any synthetic chemistry laboratory, with 10–20 non-hydrogen atoms, accuracies within 10 kJ mol^−1^ are routinely accessible. The prediction of condensed phase properties to comparable accuracy, however, is less usual. For example, gas-phase enthalpies of formation can be predicted within chemical accuracy, whereas the heat of formation of solids is not currently achievable to the same accuracy.

The condensed-phase and gas-phase standard enthalpies of formation are connected through the simple relationships

1 $${{\rm{\Delta }}}_{{\rm{f}}}H{^\circ }_{({\rm{s}})}={{\rm{\Delta }}}_{{\rm{f}}}H{^\circ }_{({\rm{g}})}-{{\rm{\Delta }}}_{{\rm{sub}}}H^\circ $$

2 $${{\rm{\Delta }}}_{{\rm{f}}}H{^\circ }_{({\rm{l}})}={{\rm{\Delta }}}_{{\rm{f}}}H{^\circ }_{({\rm{g}})}-{{\rm{\Delta }}}_{{\rm{vap}}}H^\circ $$

where ${{\rm{\Delta }}}_{{\rm{f}}}H{^\circ }_{({\rm{s}})}$, ${{\rm{\Delta }}}_{{\rm{f}}}H{^\circ }_{({\rm{l}})}$ and ${{\rm{\Delta }}}_{{\rm{f}}}H{^\circ }_{({\rm{g}})}$ are the standard enthalpy of formation of the solid, liquid and gas, respectively, and ${{\rm{\Delta }}}_{{\rm{sub}}}H^\circ$ and ${{\rm{\Delta }}}_{{\rm{vap}}}H^\circ$ are the enthalpies of sublimation and vaporization^1^, respectively.

Thus, while the gas-phase enthalpies of formation can be predicted to high accuracy with quantum mechanical methods, the prediction of enthalpies and Gibbs energies associated with phase changes is generally the realm of empirical approaches, particularly quantitative structure property relationship (QSPR) methods. There are several examples of QSPR model development for predicting enthalpies and Gibbs energies of sublimation and vaporization reported in the literature.

Modelling of the enthalpy of sublimation is an exemplar of the success of QSPR methods. The earliest attempts focused their attention on very small training sets, such as the CoMFA analysis of 30 polycyclic aromatic hydrocarbons by Welsh *et al*.^2^, and the study by Politzer *et al*.^3^ of 34 organic compounds. The squared correlation coefficient (R^2^) of these models was 0.82 and 0.95, respectively. The Politzer model used only two descriptors, the molecular surface area and a charge ‘balance parameter’ based on the surface electrostatic potential. Gharagheizi developed a model with an R^2^ of 0.97 using a larger and more diverse training set of 1079 compounds with five descriptors^4^; this model, however, has been criticized for not being generalizable and for using highly correlated descriptors^5^. Bagheri *et al*. developed a model using three descriptors and a training set including 1269 compounds with an R^2^ of 0.93^5^; this model shared two descriptors with the model developed by Gharagheizi, the topological polar surface area (*TPSA*) and the number of hydroxyl groups (*nROH*). More recently, Salahinejad *et al*. developed a model using a large heterogeneous data set of 1302 compounds with four descriptors including the fractional charged partial surface area (*FPSA*_3_), the polar surface area (*PSA*), the molecular volume (*V*) and a parameter describing the hydrophilicity (*W*1), resulting in a model with an R^2^ of 0.95^6^.

Here we review the performance of these recently reported methods for the prediction of the enthalpy of sublimation. We use a single training set to re-derive each model and compare these new models with those obtained previously using different training sets. The purpose of this review is to establish whether there is any strong dependence of each of the models on the contents of the original training dataset.

There have been significantly fewer attempts reported in the literature of QSPR models for predicting the Gibbs energy of sublimation. Perlovich and Raevsky developed a model with three descriptors, the molecular polarizability, and hydrogen bond donor and hydrogen bond acceptor factors^7^; the latter two descriptors are available within the HYBOT software package. Models for both the enthalpy and Gibbs energy of sublimation were generated using the same set of descriptors; the training sets consisted of 1316 and 686 compounds, respectively, yielding models with an R^2^ of 0.66 and 0.60, respectively.

In this study, we have applied QSPR techniques for the prediction of the Gibbs energy of sublimation. If the models for predicting the enthalpy of sublimation reliably encode information that depicts this property, it should be possible to extend these models with information describing the entropy of sublimation to estimate the Gibbs energy of sublimation; here we explore how these models perform when extended to predict the Gibbs energy of sublimation. All QSPR models were developed using the BioPPSy package^8^.

## Materials and Methods

A single set of 260 compounds with experimental values of enthalpy of sublimation was used to generate QSPR models of the enthalpy of sublimation^9^; values of Δ_sub_*H* at triple point conditions had been compiled from the DIPPR 801 database and range from 30.6 to 224 kJ mol^−1 ^^10^. It is worth noting that only 25 of the compounds in this dataset appear in the more recent compendium by Acree and Chickos^11^. For the Gibbs energies of sublimation the compilation by Perlovich and Raevsky was used^7^; this is a carefully curated dataset compiled from data obtained using different methods and at different temperatures. Notably, this dataset includes a considerable number of compounds that are normally liquids (or even gases) at 298 K and have been included in the dataset by special accounting for temperature dependencies. This set of 278 compounds was randomly divided into a training set of 244 (with Gibbs energy of sublimation ranging from 5.67 to 120.2 kJ mol^−1^) and a test set of 34 (0.92–72.2 kJ mol^−1^).

The structures of all compounds were first optimized using the MS-DOCK program^12^ to identify the global minimum conformation; this method uses the DOCK conformational search algorithm^13^ with a scoring function based on the AMBER molecular mechanics force field for estimating the energy. The structures from this search were further minimized at the B3LYP/6-31G(d) level using the GAUSSIAN-09 program^14^. The gas-phase translational and rotational entropies, *S*_trans,gas_ and *S*_rot,gas_, were obtained using GAUSSIAN-09, determined using standard statistical mechanics methods^15^.

The BioPPSy program was used to generate all QSPR models; all descriptors used in the analysis presented here are available as part of the BioPPSy package and conform to the specification in the compendium of descriptors by Todeschini and Consonni^16^. The descriptors used here include the hydrophilicity (*Hy*), molecular volume (*V*, Å^3^), first Zagreb index (*ZM1*), solvation connectivity index (*X1sol*,^*1*^*χ*^*s*^), number of hydroxyl groups (*nROH*), topological polar surface area (*TPSA*, Å^2^)^17^, Randic-type eigenvector-based index from the van der Waals weighted distance matrix (*VRv1*)^18^, reciprocal distance sum Randic-like index (*RDCHI*), surface area (*SA*, Å^2^), polar surface area (*PSA*, Å^2^) and the fractional charged partial surface area (*FPSA*_3_). The Politzer electrostatic variance parameters, ${\sigma }_{-}^{2}$ and ${\sigma }_{+}^{2}$,^3^ were calculated from the molecular electrostatic potential calculated at the B3LYP/6-31G(d) level calculated on the 0.001 a.u. electron density contour surface. From these parameters the total variance,

3 $${{\sigma }^{2}}_{{\rm{TOT}}}={{\sigma }^{2}}_{-}+{{\sigma }^{2}}_{+}$$

and balance parameter

4 $$\upsilon ={{\sigma }^{2}}_{-}{{\sigma }^{2}}_{+}{[{{\sigma }^{2}}_{{\rm{TOT}}}]}^{-2}$$

were calculated.

The SYBYL-X 2.1.1 program was used to predict the *PSA* and *FPSA*_3_. All results, including parametric equations, are reported in kJ mol^−1^. Gibbs energies of sublimation are predicted at 298 K.

### Artificial Neural Networks and Support Vector Regression

In addition to multilinear regression (MLR) we also considered Artificial Neural Networks (ANNs) and support vector regression (SVR) approaches; these have received much attention in the literature^19–26^ and are typically found to give a superior performance to MLR.

We implemented both ANNs and SVRs in BioPPSy by incorporating the machine learning package *weka*^26^. Our initial attempts, not shown, used a simple acceptance of the default parameter values given by weka. The resulting models often gave substandard fits to the training data and were unstable to testing data. However, it was reasonably straightforward to optimize these models, providing models of comparable performance compared with MLR when validating against the test data.

Optimization of the ANNs resulting in a lowering of the learning rate to 0.003 and momentum to 0.002 from the default values of 0.3 and 0.2, respectively, provided by weka. This effectively slows the learning rate for the ANN, which therefore required a corresponding amount of extra training time, measured in *epochs*; the number of epochs was increased from the default 500 to 500,000. The slower-learnt ANNs gave good fit and stable performance against non-training data.

The ANNs currently implemented in BioPPSy are all multilayer perceptrons with a single middle layer with half as many nodes as the input layer (the weka default). We have also followed the common practice of including an extra constant input “bias” node.

The ANNs were trained with a standard back propagation algorithm (available in weka), however, more stable networks exist, with neurons based on the radial basis function (RBF)^24,27^, which are more stable since they are guaranteed to reach the global minimum error surface^27^, or Bayesian neural networks^28,29^; the inclusion of such networks in BioPPSy remains part of the future development of the software.

The SVR models used in this paper use the RBF kernel that is commonly used for regression problems^22,24,25^. Although not presented here, we also investigated SVR models with the polynomial kernel, but found their performance to be consistently slightly inferior to that of MLR, and with minimal sensitivity to parameter changes. With the RBF kernel, however, SVR was capable of good fits to the training data with stable performance under validation with a testing set, but only with suitable adjustment of the gamma parameter from 0.01 to 0.1.

### Data availability

The BioPPSy program and the sublimation datasets (training and test sets) are available from https://sourceforge.net/projects/bioppsy/.

## Results and Discussion

### Enthalpy of sublimation

One of the earliest QSPR models to predict the enthalpy of sublimation was described by Politzer *et al*. using a data set composed of 34 compounds^3^. This model contains two descriptors, the molecular surface area (SA) and the product of the total variances (${\sigma }_{{\rm{tot}}}^{2}$) and the balance parameter (υ) - equation 5.

5 $${{\rm{\Delta }}}_{{\rm{s}}{\rm{u}}{\rm{b}}}H=-1.36+6.42{(\nu {{\sigma }^{2}}_{{\rm{t}}{\rm{o}}{\rm{t}}})}^{0.5}+1.82\times {10}^{-3}{(SA)}^{2}$$

The R^2^ of the model reproduced here was 0.82, compared with the original model reported by Politzer *et al*. of 0.95; the standard deviation for these two models were 13.9 and 10.5 kJ mol^−1^, respectively. The small number of compounds in the Politzer *et al*. dataset led to the model with favorable statistics.

Comparison with the original Politzer *et al*. model (equation 5a) shows close similarity with equation 5, derived using the larger dataset.

5a $${{\rm{\Delta }}}_{{\rm{sub}}}H=-9.41+8.87{(\nu {{\sigma }^{2}}_{{\rm{tot}}})}^{0.5}+1.99\times {10}^{-3}{(SA)}^{2}$$

Gharagheizi^4^ described a model based on five descriptors using a training set of 1079 compounds. Using these same descriptors with our dataset of 260 compounds we obtained the following equation (equation 6).

6 $${{\rm{\Delta }}}_{{\rm{sub}}}H=15.80-0.93(ZM{1})+14.71(X{1}sol)+14.74(nROH)+0.36(TPSA)+0.56(VRv{1})$$

The value of R^2^ of this model was 0.97, the same as the R^2^ obtained by Gharagheizi. The standard deviation of this model, 5.4 kJ mol^−1^, was also equivalent to the root mean square error (RMSE) reported by Gharagheizi, 5.5 kJ mol^−1^. The model derived by Gharagheizi (equation 6a) is very similar to equation 6 but for the coefficient for *VRv1*.

6a $${{\rm{\Delta }}}_{{\rm{sub}}}H=15.32-2.05(ZM{1})+5.18(X{1}sol)+12.37(nROH)+0.40(TPSA)+12.40(VRv{1})$$

The method for calculating the descriptor *VRv1* in BioPPSy and that used by Gharagheizi differ^18^. Thus, while the coefficient for these two descriptors differ, the descriptors themselves present the same information regarding the enthalpy of sublimation.

The third approach considered was that by Bagheri *et al*.^5^. In this model three simple parameters, *RDCHI*, *nROH* and *TPSA*, were used – equation 7.

7 $${{\rm{\Delta }}}_{{\rm{sub}}}H=22.25+9.38{(RDCHI)}^{2}+13.37(nROH)+0.42(TPSA)$$

The R^2^ of 0.96 and standard deviation of 5.1 kJ mol^−1^ calculated here compares favorably with the R^2^ and RMSE reported with Bagheri’s model of 0.93 and 9.8 kJ mol^−1^, respectively. This equation matches closely the original model described by Bagheri, equation 7a.

7a $${{\rm{\Delta }}}_{{\rm{sub}}}H=23+9{(RDCHI)}^{2}+13(nROH)+0.5(TPSA)$$

Salahinejad *et al*.^6^ showed that the enthalpy of sublimation could be adequately reproduced by a simple equation involving a single descriptor that describes the molecular volume which is accessible to and interacts with water molecules (*W1*) – equation 8

8 $${{\rm{\Delta }}}_{{\rm{sub}}}H=-16.95+0.15(W{1})$$

yielding an R^2^ of 0.90 for both training and test sets of 1042 and 260 molecules, respectively. Since the phase change from solid to liquid does not involve water, the significance of this descriptor is not immediately apparent, although it is claimed *W1* represents the hydrophilicity (or the polarizability and dispersion forces) in a molecule. We replaced *W1* with a hydrophilicity descriptor (*Hy*), to produce equation 9 with an R^2^ for the training set of 0.03.

9 $${{\rm{\Delta }}}_{{\rm{sub}}}H=82.60+8.24(Hy)$$

From this analysis, we understand *Hy* is not a suitable substitute for *W1*. Using a Bayesian feature selection approach, Salahinejad *et al*. identified three additional descriptors, *PSA* the polar surface area, *V* the water-excluded volume, and *FPSA*_3_ the fractional polar surface area, whose inclusion led to a significant improvement in their original enthalpy of sublimation model.

Using these 3 additional descriptors and replacing *W1* with *Hy*, our MLR refinement produced the following model – equation 10.

10 $${{\rm{\Delta }}}_{{\rm{sub}}}H=-\,3.84+224.54(FPS{A}_{3})+3.05(Hy)+0.57(V)+0.25(PSA)$$

The R^2^ and standard deviation of this model are 0.89 and 10.3 kJ mol^−1^, respectively, compared with the R^2^ and standard error estimation (SEE) in the Salahinejad *et al*. model of 0.95 and 7.3 kJ mol^−1^.

Removal of *Hy* from this model resulted in the following equation (equation 10a)

10a $${{\rm{\Delta }}}_{{\rm{sub}}}H=-\,7.50+279.0(FPS{A}_{3})+0.57(V)+0.26(PSA)$$

where the R^2^ and standard deviation are identical as those obtained using equation 10. We conclude that hydrophilicity does not play a significant role in describing the enthalpy of sublimation.

Finally, Mathieu generated a model using 35 group contributions yielding an R^2^ of 0.99 and an RMSE of 4.1 kJ mol^−1^ from a training set containing 814 compounds^30^. In the dataset of 260 compounds we used to create these models only 19 of the 35 group fragments were present. Using the 19 remaining groups we obtained a model with an acceptable R^2^ of 0.70, but a large standard deviation of 17.1 kJ mol^−1^. It is not unusual for models based on group contributions to have limited application beyond the molecule types included in the training set.

A comparison of the 5 models used in the prediction of the enthalpy of sublimation is presented in Table 1; the predicted heats of sublimation for all compounds for each model is provided in supporting information Table S1. The R^2^ calculated here using a training set common to the development of each model is in close agreement with the value originally obtained using 5 different datasets. The model originally developed by Bagheri *et al*. has significant appeal since the R^2^ calculated using the common set of 260 compounds matches the R^2^ calculated using their own dataset of 1269 compounds, the standard deviation is the smallest of all the models studied, and the model uses only 3 descriptors.

**Table 1:** Comparison of original models for estimating the enthalpy of sublimation and models re-derived in the current study.

	Politzer *et al*.	Gharagheizi *et al*.	Bagheri *et al*.	Salahinejad *et al*.^a^	Mathieu
Equation	5	6	7	10	
Number of descriptors	2	5	3	4	35
**Literature:** ^b^					
Dataset sizes					
(train.)	34	1079	1269	1042	814
(test)	5	269	317	260	486
R^2^ (train.)	0.95	0.97	0.93	0.95	0.99
R^2^ (test)	NA^c^	0.97	0.93	0.95	0.99
Error^d^	11.7	5.5	9.8	7.3	4.1
Re-derived here:^e^					
R^2^	0.82	0.97	0.96	0.89	0.70
Std. dev.^f^	13.9	5.4	5.1	10.3	17.1
Largest deviation^g^					
Positive	127.7	15.8	14.5	34.7	132.1
Negative	−163.9	−31.5	−20.8	−90.4	−92.9

^a^This is a modified form of the original Salahinejad *et al*. model, with the *W1* descriptor replaced by the *Hy* descriptor. ^b^Results reported in the original analysis in the literature. ^c^Not reported. ^d^Average error for the Politzer *et al*., root mean square error (RMSE) for Gharagheizi *et al*., Bagheri *et al*. and Mathieu, and standard error estimate (SEE) for Salahinejad *et al*. models in kJ mol^−1^. ^e^Results for the training set of 260 compounds from Salahinejad *et al*. re-derived here. ^f^Standard deviation in kJ mol^−1^. ^g^Deviation from experiment in kJ mol^−1^.

The largest deviations from experiment for each model, both positive and negative, are presented in Table 1. The Politzer and Mathieu models performed particularly poorly in the prediction for bis-2-hydroxyethyl-terephthalate (127.7 and 132.1 kJ mol^−1^) and di-n-butyl-sulfide (−163.9 and −92.9 kJ mol^−1^), respectively. The enthalpy of sublimation of 2,6-di-tert-butyl-4-methylphenol was poorly predicted by all methods, with errors of 35.2, 15.4, 12.8 and 72.5 kJ mol^−1^ for equations 5, 6, 7 and 10, respectively. For the Bagheri *et al*. model, the difference from experiment for the entire set of 260 compounds ranged from 14.5 (adiponitrile) to −20.8 (2,3,5-trimethyl-3a,4,7,7a-tetrahydro-1*H*-4,7-methanoindene) kJ mol^−1^. Thus, within its domain of applicability, this method should produce estimates of the enthalpy of sublimation with an accuracy of approximately 20 kJ mol^−1^.

There have been two other attempts to develop models for the prediction of the enthalpy of sublimation^2,31^. These studies focused their attention on specific classes of compounds (polyaromatic hydrocarbons and explosives) and are unlikely to be extensible beyond those classes.

### Gibbs energy of sublimation

None of the models used to predict the enthalpy of sublimation could be used to train a model suitable to predict the Gibbs energy of sublimation. Each of the models described above were trained against the dataset of 278 Gibbs sublimation energies from Perlovich and Raevsky; the R^2^ and standard deviation from each model is presented in Table 2. Using the descriptors from the Politzer *et al*. enthalpy of sublimation model we produced a very poor model for the prediction of Gibbs energy of sublimation, R^2^ of 0.23. Using the group parameters in Mathieu’s enthalpy model, the R^2^ was 0.25. Using the descriptors from the Gharagheizi, Bagheri *et al*., and Salahinejad *et al*. enthalpy of sublimation models created models for predicting the Gibbs energy that were also unsatisfactory, with an R^2^ all less than 0.60. Thus, without descriptors that capture information regarding the entropy of sublimation, the models that adequately describe the enthalpy of sublimation cannot be repurposed to describe the Gibbs energy of sublimation without appending terms that encode the entropy.

**Table 2:** Comparison of models for estimating the Gibbs energy of sublimation.

Model	R^2^ (train.)^a^	Std. dev.^b^ (kJ mol^−1^)
Politzer *et al*.	0.23	17.2
Gharagheizi	0.55	13.1
Bagheri *et al*.	0.58	12.7
Salahinejad *et al*.	0.59	12.5
Mathieu	0.25	17.0

^a^R^2^ for the training set of 278 compounds from Perlovich and Raevsky. ^b^Standard deviation.

Considering the Gibbs-Helmholtz equation for the Gibbs energy of sublimation

11 $${{\rm{\Delta }}}_{{\rm{sub}}}G^\circ ={{\rm{\Delta }}}_{{\rm{sub}}}H^\circ -T.{{\rm{\Delta }}}_{{\rm{sub}}}S^\circ $$

it should be possible to predict the Gibbs energy of sublimation from knowledge of the enthalpy and entropy of sublimation. The entropy of sublimation depends on the molecular interactions between the molecules, and their influence on the order in the solid. Thus, it should be possible to model the entropy of sublimation with molecular descriptors that reflect the different types of non-covalent interactions in solids, namely ionic, hydrogen bonding and van der Waals. Applying different QSPR models for calculating the enthalpy of sublimation, we generated QSPR models for the prediction of the Gibbs energy of sublimation.

Initially we applied four descriptors from the Salahinejad *et al*. model for predicting the enthalpy of sublimation model (*Hy*, *V*, *PSA*, *FPSA*_3_) – where *Hy* was used as a substitute for the *W1* descriptor - and the gas-phase entropies for translation and rotation, *S*_trans,gas_ and *S*_rot,gas_, to build a QSPR model. The R^2^ value of the model based on the training set for this model was 0.60. Outliers in this model were identified to contain two characteristic features, conjugated systems and zwitterionic compounds. Thus, two descriptors, the number of fused rings in the molecule (*R*_*fused*_) and the zwitterionic nature of the molecule (*Zwit*)^32^, were included into the model. The R^2^ of the training set improved to 0.71, indicating these two new descriptors contributed constructively to the improved model. The value of standard deviation of this model was 10.3 kJ mol^−1^. The resulting relationship is shown in equation 12.

12 $$\begin{array}{rcl}{{\rm{\Delta }}}_{{\rm{sub}}}G^\circ  & = & 10.47+5.70(Hy)+0.15(V)+0.10(PSA)+134.95(FPS{A}_{3})\\  &  & +\,2.24({S}_{\mathrm{trans},\mathrm{gas}})+0.26({S}_{\mathrm{rot},\mathrm{gas}})+4.58({R}_{{\rm{fused}}})+18.51(Zwit)\end{array}$$

**Figure 1:**
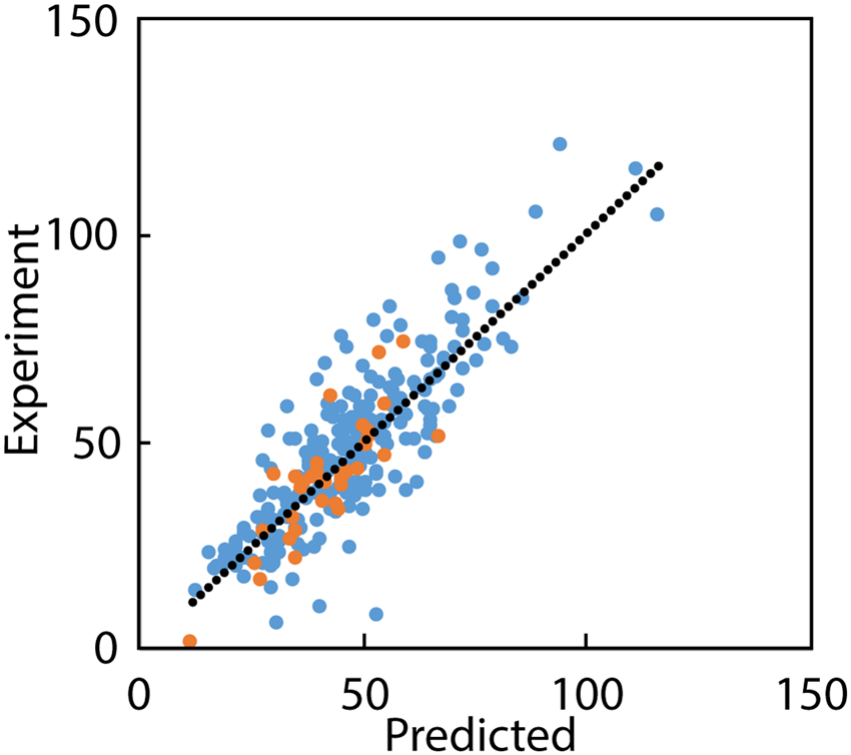
Comparison of predicted values of Gibbs energy of sublimation versus experimental. Training set (blue) and test set (red) generated by the MLR-based model, equation 13. Energies are in units of kJ mol^−1^.

We found the gas-phase entropy descriptors, *S*_trans,gas_ and *S*_rot,gas_, could be discarded to produce a new model using just six descriptors, resulting in a robust model with an R^2^ for the training set of 0.71, an R^2^ of the test set of 0.66, and a standard deviation of 10.5 kJ mol^−1^ (equation 13). Notably, inclusion of the gas-phase entropy descriptors, *S*_trans,gas_ and *S*_rot,gas_, did not significantly improve any model developed here. A plot of predicted values of Gibbs energy of sublimation versus experimental for the training and test sets is presented in Fig. 1.

13 $${{\rm{\Delta }}}_{{\rm{sub}}}G^\circ =-10.55+5.76(Hy)+0.19(V)+0.11(PSA)+121.87(FPS{A}_{3})+4.68({R}_{fused})+17.42(Zwit)$$

The hydrophobicity descriptor, *Hy*, could be removed from equation 13 with little effect on R^2^ or the standard deviation (0.71 and 9.1 kJ mol^−1^, respectively), consistent with its lack of influence in the corresponding model for the enthalpy of sublimation (equation 10a). However, without *Hy* the R^2^ and standard deviation for the test set was 0.62 and 12.6 kJ mol^−1^, respectively, significantly worse than the model with *Hy* included (0.66). The improved performance of the model including the *Hy* descriptor suggests inclusion of a descriptor that encodes hydrophobicity is necessary to avoid overfitting the model, and therefore warrants its inclusion in the final model.

In the second approach, we applied the Politzer *et al*. enthalpy of sublimation QSPR model as the underlying set of descriptors ((*SA*)^2^ and ${{({\rm{\nu }}{\rm{\sigma }}}_{{\rm{tot}}}^{2})}^{0.5}$) supplemented with the two entropy-related descriptors, *R*_*fused*_ and *Zwit*. Using these four descriptors, the following equation (equation 14) was obtained,

14 $${{\rm{\Delta }}}_{{\rm{sub}}}G^\circ =10.31+2.82{({{{\rm{\nu }}{\rm{\sigma }}}^{2}}_{{\rm{tot}}})}^{0.5}+1.50\times {10}^{-4}{(SA)}^{2}+7.25({R}_{fused})+22.45(Zwit)$$

where the R^2^ for the training set was 0.51, the R^2^ for the test set was 0.29, and the standard deviation was 12.6 kJ mol^−1^. The small value of R^2^ indicates this model is neither particularly robust nor predictive.

In the third approach, the multivariate model using seven parameters, *RDCHI*, *nROH*, *TPSA*, *R*_*fused*_, and *Zwit* was obtained (equation 15) with an R^2^ for the training set of 0.66, an R^2^ for the test set of 0.54, and a standard deviation of 10.1 kJ mol^−1^.

15 $$\begin{array}{rcl}{{\rm{\Delta }}}_{{\rm{sub}}}G^\circ  & = & 4.72+4.23{(RDCHI)}^{2}+2.50(nROH)+0.26(TPSA)\\  &  & +\,3.60({R}_{fused})+22.50(Zwit)\end{array}$$

In the final approach considered here, the underlying model was the enthalpy of sublimation model of Bagheri *et al*.^5^. Seven descriptors (*ZM1*, *X1sol*, *nROH*, *TPSA*, *VRv1*, *R*_*fused*_ and *Zwit*) were considered; the R^2^ of the training and test sets were 0.67 and 0.56, respectively, and the standard deviation was 10.1 kJ mol^−1^ (equation 16).

16 $$\begin{array}{rcl}{{\rm{\Delta }}}_{{\rm{sub}}}G^\circ  & = & 3.73-0.50(ZM1)+7.72(X{1}sol)+3.50(nROH)+0.23(TPSA)\\  &  & +\,0.18(VRv{1})+4.89({R}_{fused})+22.04(Zwit)\end{array}$$

A comparison of the various models developed here is presented in Table 3; the predicted Gibbs energy of sublimation for all compounds for each model is provided in supporting information Table S2. A y-randomization test of these four models (equations 13–16) yielded R^2^ values of 0.01–0.06 and standard deviations of 19.1–19.5 kJ mol^−1^, indicating minimal effect of any chance correlation in the refined models. The model described by equation 13 is a good compromise between performance and number of descriptors.

**Table 3:** Comparison of MLR models for estimating the Gibbs energy of sublimation.

Equation	R^2^	Std. dev.^a^	Largest deviation^a^		R^2^	Std. dev.^a,b^
	(train.)		Positive	Negative	(test)	
13	0.71	10.5	28.8	−45.4	0.66	8.6
14	0.51	12.6	50.2	−38.3	0.29	12.4
15	0.66	10.1	31.3	−37.5	0.54	9.9
16	0.67	10.1	29.9	−33.3	0.56	9.7

^a^kJ mol^−1^. ^b^Standard deviation for the test set.

The initial dataset of 278 compounds was partitioned into ten different training and test sets to explore the dependency of the performance of the model on the separation scheme. For the model described by equation 13, ten different partitioning attempts yielded R^2^ for the training set between 0.71 and 0.73, between 0.53 and 0.72 for the test set, and standard deviations in the range 9.9 to 10.4 kJ mol^−1^. Similar variation was observed for the other models.

The predicted Gibbs energy of sublimation differed significantly from the experimental value for several compounds in all 4 models; 1-amino-2-methyl-9,10-anthraquinone (errors in the range 26.1–38.1 kJ mol^−1^), perfluorohexamethylprismane (−27.8–−45.4 kJ mol^−1^), *N*-acetyl-L-isoleucineamide (23.5–30.8 kJ mol^−1^), and 2-nitro-benzonitrile (−25.1–−34.0 kJ mol^−1^). In the model created by Perlovich and Raevsky these compounds exhibited errors of 28.0, −17.8, 7.0, and −18.8 kJ mol^−1^, respectively. Thus, it appears these compounds represent systems that are challenging for QSPR models to describe accurately. The range of values of the descriptors in each of the models described in Table 3 are listed in Table 4. These limits define the domain of the applicability of each method^33^.

**Table 4:** Bounding box definitions of domain of applicability for models for estimating the Gibbs energy of sublimation.

Descriptor	Minimum	Maximum
*Hy*	−6.5 × 10^−4^	2.1
*V*	49.5	460.7
*PSA*	0	270.1
*FPSA*_3_	0	9.9 × 10^−2^
*R*_*fused*_	0	9
*Zwit*	0	1
*SA*	29.0	505.7
(νσ^2^_tot_)^0.5^	4.3 × 10^−1^	10.5
*RDCHI*	1.2	4.4
*nROH*	0	3
*TPSA*	0	92.4
*ZM1*	6	216
*X1sol*	1.4	16.9
*VRv1*	3.7	260.8

The model described by equation 13 fulfills the criteria of a useful model, an R^2^ of the test set greater than 0.6 and low RMSE (or standard deviation) of the test set predictions^34^.

More recently McDonagh *et al*. developed models for predicting the enthalpy, entropy and Gibbs energy of sublimation^35^. Experimental data for the enthalpy, entropy and Gibbs energy were available for all 158 compounds used in the training set. Using only 2D descriptors, the partial least squares (PLS) method yielded an R^2^ of 0.65 and 0.76 for the enthalpy and Gibbs energy, respectively. For the enthalpy of sublimation, the Salahinejad *et al*. model presented in equation 10 performed significantly better than any of the models presented by McDonagh *et al*., despite McDonagh *et al*. including a larger number of descriptors and using a smaller training set. For the Gibbs energy of sublimation, the PLS model of McDonagh *et al*. performs slightly better than that model described here in equation 13, although the McDonagh *et al*. model has the advantage of a larger number of descriptors and smaller training set. The McDonagh *et al*. models included only a single descriptor in common with the models presented here, the TPSA.

Presented in Table 5 is presented the performance of two non-linear regression algorithms, ANN and SVR, using the same descriptors used in equations 13–16; the predicted Gibbs energy of sublimation for all compounds for each model is provided in supporting information Tables S3 and S4. Multivariate regression with ANN using the descriptors included in equation 13 produced a model with a significantly improved R^2^, 0.80, compared with the MLR R^2^ of 0.71. The improvement in the R^2^ of the test set, however, was significantly more modest, 0.63 using ANN over 0.62 from MLR, indicating the predictability of the ANN model is not significantly better than the MLR model. The small R^2^ for the training set, and a negative R^2^ for the test set, using ANN with the descriptors in equation 14 indicates a poor model that is not predictive. While the use of SVR produces a slightly more predictive model (R^2^ of the test set of 0.26), the model retains very little value. Application of either ANN or SVR with the descriptors in equations 15 or 16 improves slightly the quality of the models over MLR – improvements in R^2^ for both training and test sets are roughly 0.05. Again, the model described by equation 13 fulfills the criteria of a useful model^34^.

**Table 5:** Comparison of ANN and SVR models for estimating the Gibbs energy of sublimation.

Equation	13	14	15	16
**Artificial Neural Networks**				
R^2^ (train.)	0.80	0.59	0.71	0.73
Std. dev.^a^	8.9	16.6	9.4	9.6
Largest deviation^a^				
Positive	28.1	38.8	40.2	36.9
Negative	−31.3	−41.3	−32.8	−33.0
R^2^ (test)	0.63	−0.32	0.59	0.57
Std. dev.^a,b^	8.7	11.5	9.3	9.5
**Support Vector Regression**				
R^2^ (train.)	0.77	0.51	0.70	0.71
Std. dev.^a^	9.2	12.4	9.8	9.6
Largest deviation^a^				
Positive	38.5	38.0	37.3	39.5
Negative	−36.5	−53.3	−33.0	−34.8
R^2^ (test)	0.61	0.26	0.56	0.58
Std. dev.^a,b^	9.0	11.9	9.8	9.5

^a^kJ mol^−1^. ^b^Standard deviation for the test set.

## Conclusion

In this study, we have reproduced several QSPR models reported previously for the prediction of the enthalpy of sublimation. We have trained each model using a single consistent training set. From this comparison, we observe that all QSPR models based on molecular descriptors perform well. In contrast, the one model we examined using a fragment-based approach, did not perform well.

We also developed several QSPR models for estimating the values of the Gibbs energy of sublimation with simple descriptors in the BioPPSy package. Models that performed well in predicting the enthalpy of sublimation could not be trained to predict the Gibbs energy of sublimation with any confidence. Inclusion of two descriptors that describe intermolecular interactions, the number of fused rings and the potential to form a zwitterion, could be used to improve these models. The preferred model based on MLR refinement has six descriptors, hydrophilicity, molecular volume, polar surface area, fractional charged partial surface area, the number of fused rings and the potential to form a zwitterionic species, with a squared correlation coefficient of 0.71 and standard deviation of 10.6 kJ mol^−1^. ANN refinement using these same descriptors produced a model with significantly improved R^2^ and standard deviation, however, the predictability, as gauged by the calculated R^2^ for the test set, was not significantly improved.

## Electronic supplementary material

Comparison of experimental and predicted enthalpies and Gibbs energies of sublimation
