# Supplementary material for: Predicting the Enthalpy and Gibbs Energy of Sublimation by QSPR Modeling
Source: Sci Rep. 2018 Jun 27;8:9779. doi: 10.1038/s41598-018-28105-6 (PMC6021403; doi:10.1038/s41598-018-28105-6)
Supplement: Supplementary file 1 — Comparison of experimental and predicted enthalpies and Gibbs energies of sublimation [file 41598_2018_28105_MOESM1_ESM.pdf]

Predicting the Enthalpy and Gibbs Energy of Sublimation by QSPR Modeling

Nastaran Meftahi, Michael L. Walker, Marta Enciso, Brian J. Smith✉

La Trobe Institute for Molecular Science, La Trobe University, Melbourne, Victoria 3086, Australia.

Table S1. Comparison of experimental and predicted enthalpies of sublimation (kJ mol^{-1}).

	Expt	Eq. 5	Eq. 6	Eq. 7	Eq. 10	Mathieu
1,1,2-trichloroethane	47.9	70.9	54.7	40.7	43.0	51.5
1,1,2-triphenylethane	116.0	118.2	124.5	126.5	111.9	63.0
1,1-dichlorotetrafluoroethane	34.0	49.7	49.7	44.8	47.7	37.8
1,2,3,5-tetraethylbenzene	90.3	90.1	89.0	78.6	86.1	66.0
1,2,3-trimethylindene	68.7	73.7	68.9	72.5	75.1	60.8
1,2,4-triethylbenzene	79.6	78.1	77.8	72.8	76.4	63.6
1,2,4-trimethyl-5-ethylbenzene	72.2	72.2	66.5	65.6	72.3	50.4
1,2-benzenedicarboxylic-acid-heptyl-nonyl-ester	207.0	218.7	202.0	207.1	186.7	163.1
1,2-dibromododecane	108.0	108.6	123.2	94.4	103.1	97.4
1,2-dimethyl-3-propylbenzene	73.5	72.9	69.9	68.4	72.6	58.4
1,3,5-triethylbenzene	80.4	78.4	77.5	73.1	78.4	63.6
1,3-propanediamine	67.6	73.2	60.3	65.7	83.9	78.4
1,4-butanediol	91.7	84.4	92.5	92.7	86.3	107.5
1,4-dichlorobutane	55.9	66.3	61.3	48.7	49.3	69.5
1,4-dichloro-trans-2-butene	54.2	67.3	61.3	48.7	51.6	56.5
1,4-dioxane	51.5	59.0	51.8	60.5	51.3	89.6
1,6-hexanediol	103.0	89.1	106.0	103.7	92.4	117.9
1-bromobutane	47.0	57.0	54.5	43.6	44.8	64.4
1-bromoheptane	65.7	69.0	74.9	59.7	64.1	80.0
1-decyne	79.5	44.2	74.8	71.9	76.0	66.4
1-eicosene	149.0	152.7	142.6	147.1	138.9	132.9
1-hexene	47.6	54.7	47.7	48.7	49.8	60.0
1-methyl-3-methylethoxy-benzene	75.8	74.7	71.9	74.1	73.3	55.6
1-nitropropane	62.7	70.2	59.2	63.9	65.1	79.3
1-nonene	67.6	66.7	68.0	65.6	68.6	75.6
1-pentene	41.2	51.9	40.9	43.6	44.3	54.8
1-phenylnaphthalene	88.6	89.1	97.7	102.1	92.5	56.4
1-undecene	81.1	77.6	81.6	78.3	81.3	86.0
2,2,3-trimethylbutane	43.3	51.0	39.7	43.6	48.2	52.4
2,2,4-trimethyl-1,3-pentanediol-diisobutyrate	122.0	144.2	131.1	121.1	124.3	93.0
2,2-dichloro-1,1,1-trifluoroethane	38.2	98.4	50.9	43.6	45.9	117.5
2-(2-hexoxyethoxy)ethanol	122.0	56.1	124.2	121.9	111.2	37.7
2-(2-butoxyethoxy)ethanol	108.0	101.6	110.7	108.2	99.5	127.9
2,3,3,4-tetramethylpentane	48.8	60.0	50.7	49.9	57.0	51.0
2,3,3-trimethyl-1-butene	40.8	58.1	39.7	43.6	51.6	51.5
2,3,4-trimethylpentane	52.2	55.4	50.0	49.4	56.5	46.7
2,3-dichloro-1-propanol	75.0	76.0	79.0	67.0	65.9	75.7
2,3-dimethyl-2-butene	43.7	53.3	39.6	42.7	48.2	56.6
2,3-dimethyloctane	69.2	66.0	67.4	63.8	71.6	68.9
2,3-dimethylpentane	49.4	51.5	47.1	47.1	52.3	53.3
2,4,4-trimethylhexane	54.5	60.5	53.9	53.3	59.1	62.8
2,4-dimethylhexane	55.4	55.9	53.6	52.6	57.3	58.5
2,4-xylenol	82.7	78.7	77.7	80.9	75.8	65.9
2,6,8-trimethyl-4-nonanone	87.6	96.8	86.0	82.2	86.0	75.9
2,6-diethylnaphthalene	87.3	85.1	86.1	87.2	88.7	61.2
2,6-dimethyl-4-heptanol	85.7	88.2	85.3	82.9	74.0	75.1
2,6-Di-tert-butyl-4-methylphenol	86.9	122.1	102.3	99.7	159.4	113.8
2,6-naphthalenedicarboxylic-acid	149.0	76.7	149.0	151.9	143.3	101.8
2-aminoethoxyethanol	91.4	69.9	89.9	91.0	95.8	100.9
2-butoxyethanol	86.0	121.9	87.0	85.6	78.6	124.6
2-butyl-1-decanol	130.0	74.6	128.3	120.1	110.2	73.9
2-butyne-1,4-diol	106.0	64.5	92.5	92.7	91.9	83.0
2-cyclohexene-1-one	55.6	85.0	54.6	61.9	58.6	93.4
2-ethyl-1-hexanol	86.9	62.7	87.6	82.0	77.3	69.4

2-ethyl-1-hexene	58.3	71.6	57.8	55.6	59.9	77.4
2-ethyl-2-hexenal	72.4	68.0	71.5	67.2	75.0	53.2
2-ethyl-m-xylene	67.2	76.0	63.1	62.7	65.3	85.1
2-heptanone	67.4	81.5	63.3	63.7	67.7	85.9
2-hydroxypropyl-acrylate	90.4	66.8	92.3	91.5	90.5	74.6
2-methyl-1-octene	65.0	64.2	63.8	62.4	67.2	67.0
2-methyl-2-pentenal	60.9	65.7	57.2	57.4	64.3	53.6
2-methylbenzofuran	65.5	47.9	65.7	74.9	68.0	69.3
2-methyl-dodecan-1-ol	123.0	69.1	120.8	116.4	110.1	74.4
2-methylhexanal	68.3	97.4	64.0	62.8	69.5	106.9
2-methyloctanoic-acid	104.0	47.6	102.8	99.3	97.4	59.8
2-methylpentane	46.8	69.3	43.5	45.6	47.4	68.2
2-methyl-thiaindan	73.4	56.0	78.0	80.0	68.1	69.4
3,3-dimethylhexane	50.2	51.7	51.3	50.6	53.8	64.2
3,3-dimethylpentane	45.3	71.3	44.5	45.5	47.3	46.1
3,4-dichloro-1-butene	50.1	76.9	56.6	45.0	50.0	90.4
3-amino-1-propanol	76.2	67.8	73.0	76.7	83.7	82.0
3-hydroxy-2-methyl-propionaldehyde	76.0	51.8	72.8	74.3	73.7	43.0
3-methyl-1-butene	35.2	58.1	36.7	40.7	42.4	53.4
3-methyl-1-hexene	51.3	75.9	51.0	50.1	54.4	82.1
3-methyl-3-pentanol	66.2	56.0	66.9	67.5	54.4	70.2
3-methylheptane	59.4	51.6	57.8	55.6	59.7	65.0
3-methylhexane	52.8	61.1	51.0	50.1	53.3	75.4
3-methyloctane	65.7	58.9	64.5	61.4	66.1	53.6
3-methylthiophene	48.5	69.0	61.5	62.2	52.8	85.1
4-heptanone	67.7	97.5	64.0	62.6	62.4	82.3
4-hydroxyacetophenone	94.5	79.1	90.7	93.7	93.0	93.7
4-hydroxybutyraldehyde	79.3	79.9	76.3	77.9	78.2	48.2
4-isobutylstyrene	70.8	62.5	76.1	76.0	78.6	58.7
4-methyl-1-heptene	67.6	78.6	57.8	55.4	60.6	71.2
4-methyl-2-pentanol	70.3	56.2	68.4	69.9	61.1	70.2
4-methylheptane	59.5	52.8	57.8	55.4	59.5	46.9
4-methyl-trans-2-pentene	46.1	81.1	43.5	45.6	48.8	95.6
4-nonanone	78.2	73.5	77.5	74.3	78.2	90.3
4-octanone	73.4	70.9	70.8	68.3	71.8	68.4
5-hexen-2-one	56.8	98.0	56.5	58.1	62.4	126.3
6-hydroxyhexanoic-acid	115.0	70.1	114.8	113.6	107.1	53.8
acenaphthalene	69.4	97.5	70.5	80.4	66.6	95.6
acetoacetanilide	105.0	80.1	99.1	102.0	103.8	89.6
acetomethoxane	85.9	38.8	88.2	91.5	85.9	56.0
acetone-cyanohydrin	76.0	36.6	67.7	73.5	72.8	50.8
acrylonitrile	43.1	36.6	42.8	49.0	57.6	50.8
adiponitrile	94.4	91.4	78.6	79.9	87.0	103.2
alpha-hydroxyisobutyric-acid	91.4	65.8	90.7	94.9	84.5	57.9
alpha-phellandrene	59.7	65.6	62.1	64.4	66.6	59.8
anisole	60.4	93.9	59.2	63.3	61.2	83.5
anthraquinone	103.0	54.8	104.1	108.4	102.7	56.4
azelaic acid	146.0	76.4	143.9	143.6	171.1	53.4
benzene	45.3	97.2	45.0	52.7	47.3	81.3
benzotrichloride	68.5	69.5	73.4	62.6	68.4	57.7
benzyl-benzoate	109.0	69.6	111.2	117.6	107.6	70.2
benzyl-chloride	60.6	73.9	62.7	59.4	59.1	74.4
benzyl-ethyl-ether	72.8	125.0	72.8	75.0	71.3	147.0
benzyl-formate	81.6	57.9	79.0	82.2	83.5	51.6
bis-2-hydroxyethyl-terephthalate	174.0	46.3	178.5	175.4	157.9	41.9
bromobenzene	59.0	104.6	59.5	54.6	54.3	69.1
bromotrifluoroethylene	43.1	54.5	50.8	42.7	43.4	46.3
chloroform	38.5	89.3	46.0	36.3	36.7	69.3
cis-1,3-dimethylcyclopentane	46.2	64.0	44.9	52.5	49.0	51.0
cis-1-propenylbenzene	62.7	65.8	62.6	65.0	62.5	57.6

cis-crotonaldehyde	50.7	82.8	47.1	50.9	56.7	53.8
cis-stilbene	88.8	95.4	91.2	97.9	94.2	115.4
citraconic-acid	113.0	39.9	113.7	113.5	107.0	77.2
cyclobutane	33.6	46.8	31.5	46.3	31.8	65.4
cyclopropyl-cyanide	44.9	88.8	44.2	59.1	56.2	90.5
diacetone-alcohol	77.1	66.0	74.5	78.3	72.7	56.4
diallyl-sulfide	62.6	77.3	73.3	64.8	61.1	56.4
dibenzothiophene	90.3	51.7	95.5	98.4	82.5	43.1
dichlorofluoromethane	32.9	90.6	40.4	36.3	34.6	117.2
diethanolamine	96.7	66.4	103.9	103.1	99.7	77.2
diethyl-disulfide	62.4	78.8	87.8	70.1	53.1	112.3
diethylene-glycol-monopropyl-ether	101.0	66.7	103.9	101.7	91.8	86.3
diethylethanolamine	82.0	81.8	82.1	77.8	75.7	98.1
diethyl-maleate	97.9	76.0	100.6	97.4	91.6	105.9
diethyl-malonate	92.3	79.9	93.8	90.9	91.8	111.1
diethyl-succinate	98.9	79.8	100.6	97.4	96.1	72.0
diisobutyl-ketone	59.7	65.6	69.1	68.1	70.0	57.8
diisopropylamine	57.3	88.8	50.6	53.0	59.3	114.0
dimethyl-1,4-cyclohexanedicarboxylate	102.0	110.1	106.7	105.1	108.7	90.3
dimethyl-2,6-naphthalenedicarboxylate	125.0	36.6	126.1	126.8	127.9	50.8
dimethylacetylene	36.7	48.1	34.1	38.9	37.9	57.0
dimethyl-ether	33.0	75.9	30.7	38.7	37.1	87.7
dimethyl-maleate	97.9	71.4	87.1	85.7	85.7	95.5
dimethylmalonate	80.8	74.2	80.3	79.7	81.2	90.0
di-n-butyl-sulfide	80.6	244.5	86.8	76.4	70.0	173.5
di-n-nonyl-phthalate	224.0	126.8	215.5	225.7	199.3	133.4
di-n-octylamine	138.0	203.0	126.7	127.3	125.5	149.2
dinonylphenol	202.0	76.4	187.9	189.4	167.3	98.6
di-n-pentyl-ether	85.7	65.0	84.9	82.2	81.9	81.3
di-n-propylamine	63.7	74.7	59.0	59.1	62.9	87.6
di-n-propyl-disulfide	75.2	94.7	101.3	81.1	68.8	72.4
diphenyl-disulfide	112.0	85.9	129.5	119.3	95.6	94.0
dipropylene-glycol-monoethyl-ether	99.4	174.2	103.7	99.0	95.1	154.8
dodecanal	106.0	99.5	101.3	99.3	103.5	112.2
dodecyl-bromide	101.0	98.1	108.8	92.0	96.4	106.0
ethanol	51.1	77.5	49.8	56.8	52.9	73.9
ethyl-acetate	56.1	56.8	53.1	56.7	58.1	75.7
ethyl-acrylate	60.2	63.4	60.6	61.3	64.5	69.4
ethylbenzene	57.6	61.0	55.9	59.4	58.8	58.8
ethyl-benzoate	82.6	82.8	82.7	83.4	83.0	78.5
ethyl-isoamyl-ketone	69.9	74.8	66.6	65.6	70.8	78.6
ethyl-isovalerate	71.3	67.9	69.9	69.2	69.6	79.6
ethyl-mercaptan	40.2	56.2	48.6	51.2	50.9	47.4
ethyl-vanillin	101.7	91.5	109.1	107.0	102.7	86.7
fluorene	82.1	73.8	77.3	86.4	77.8	61.6
formaldehyde	32.9	60.5	28.5	38.8	46.7	56.3
formanilide	80.4	83.0	73.4	77.3	85.5	72.7
fumaronitrile	66.4	47.4	65.0	68.8	84.1	44.4
gamma-terpinene	59.9	64.7	62.1	64.4	66.3	68.7
glutaronitrile	81.0	38.8	71.8	74.2	81.7	56.0
glycerol	109.0	98.5	111.5	111.1	103.7	113.7
halothane	43.6	54.3	56.5	43.6	48.4	36.7
hexamethyleneimine	60.8	62.4	56.4	61.6	57.4	60.8
isobutylbenzene	66.8	69.1	65.2	67.8	66.5	57.4
isobutyl-formate	65.5	64.5	59.9	62.0	69.4	70.2
isophthaloyl-chloride	96.4	92.4	96.1	85.8	95.3	75.8
isopropyl-myristate	141.0	150.0	137.7	139.6	132.4	131.6
l-menthol	85.0	87.9	88.1	88.1	80.9	84.5
l-phenylalanine	121.0	110.4	113.9	116.1	118.2	95.0
lysine	122.0	107.9	115.4	115.1	126.3	113.8

malononitrile	63.2	36.6	58.3	63.8	76.5	50.8
mesityl-oxide	57.2	64.2	52.3	55.2	58.7	65.9
methyl-chloride	30.6	53.0	31.9	31.6	25.4	49.7
methylcyclopentadiene-dimer	57.2	69.4	63.9	78.0	71.1	75.8
methylethanolamine	71.5	79.8	68.0	70.7	72.3	88.9
methyl-ethyl-ketone	47.9	62.7	42.9	47.9	50.5	69.5
methyl-lactate	80.1	74.9	79.1	80.2	79.1	87.1
methyl-para-toluate	81.7	75.4	79.2	80.5	83.7	70.5
methyl-phenyl-sulfide	68.9	66.6	75.8	70.1	61.7	61.6
methyl-vinyl-ether	37.5	59.3	37.5	42.9	43.1	50.6
monochlorobenzene	54.0	60.7	53.9	54.6	52.1	52.5
n-butyl-methacrylate	77.1	73.2	77.0	75.0	79.9	83.9
n-butyl-stearate	188.0	191.1	175.8	186.9	168.6	169.4
n-decyl-acetate	110.0	98.2	107.3	106.8	95.2	117.4
n-decylbenzene	112.0	108.7	110.1	113.8	106.5	100.4
n-decylcyclohexane	117.0	101.4	110.1	113.8	104.9	127.9
n-decyl-formate	109.0	106.5	104.7	103.2	107.5	113.2
docosane	151.1	103.3	156.2	164.7	241.5	99.5
n-dodecyl-mercaptan	109.0	149.2	116.2	108.5	110.1	144.4
n-eicosane	152.3	75.1	142.6	147.1	138.5	68.5
n-ethylaniline	71.6	160.6	67.2	70.1	73.4	149.6
n-heneicosane	141.8	74.6	149.4	155.8	144.9	96.5
n-hexyl-acetate	82.1	71.8	80.2	79.7	81.9	92.4
n-hexyl-formate	80.6	74.0	77.6	76.8	83.1	63.3
n-methylaniline	65.5	78.5	60.4	64.5	69.3	60.8
N,N-diethylaniline	73.7	74.1	75.0	72.2	71.6	75.9
N,N-diethylhydroxylamine	68.4	68.7	67.8	68.4	62.6	50.4
N,N-dimethylaniline	62.3	65.5	60.0	63.0	64.4	56.9
N,N-dimethylformamide	50.4	61.1	44.1	49.3	57.1	87.2
n-nonane	70.0	83.9	68.0	65.6	67.5	83.9
n-nonyl-mercaptan	87.4	85.0	95.8	88.3	91.1	106.9
n-octyl-acetate	95.7	80.9	93.7	92.7	94.4	96.6
nonanal	84.5	121.7	81.0	79.1	84.6	150.2
n-nonylamine	89.2	73.2	84.4	82.9	92.5	68.5
n-pentadecylbenzene	150.0	151.6	144.0	155.7	138.5	126.4
n-pentane	43.4	44.2	40.9	43.6	41.8	66.4
n-pentyl-acetate	75.5	69.8	73.4	73.5	75.7	91.3
n-pentylamine	61.9	69.5	57.3	59.7	69.3	77.6
n-propyl-acetate	62.6	60.2	59.9	62.0	63.7	80.9
n-propyl-chloride	39.2	56.0	41.0	38.9	36.6	60.1
n-propyl-mercaptan	46.8	58.1	55.3	55.4	55.9	52.6
n-propyl-methacrylate	70.5	69.1	70.3	69.0	73.7	78.7
n-propyl-n-butyrate	75.6	69.9	74.1	72.2	73.6	91.3
n-propyl-propionate	68.8	64.0	67.4	66.7	69.3	86.1
n-tridecylbenzene	134.0	132.5	130.5	138.4	125.6	116.0
n-undecane	84.0	72.6	81.6	78.3	80.4	97.6
o-chlorotoluene	59.7	63.8	57.6	56.4	56.8	49.7
o-ethylstyrene	66.6	69.8	67.0	65.4	66.8	49.6
o-tolualdehyde	71.1	76.6	65.7	68.2	68.9	60.2
p-cumylphenol	111.0	108.3	114.8	115.9	107.8	74.5
p-diethylbenzene	68.7	68.7	66.7	67.0	70.4	61.2
p-diisopropylbenzene-hydroperoxide	103.0	99.8	106.3	104.3	98.4	76.5
phenetole	66.6	67.1	66.0	68.9	65.5	65.0
phenylhydrazine	77.8	76.0	70.1	75.5	90.7	69.3
p-menthane-hydroperoxide	89.3	96.2	95.9	95.9	86.5	101.6
p-phenylenediamine	70.7	81.9	71.0	79.1	99.0	62.8
propanal	45.1	56.9	40.4	46.2	48.6	65.4
p-terphenyl	116.0	101.3	110.9	119.5	106.2	56.4
p-tert-butylcatechol	99.3	97.6	110.6	111.7	102.7	86.9
p-tert-butyl-ethylbenzene	66.4	78.2	69.7	70.8	73.7	59.0

p-tolualdehyde	71.4	73.0	65.4	69.2	73.0	60.2
P-toluic acid	88.7	94.8	90.7	93.7	92.0	82.3
pyridazine	57.4	77.1	54.5	63.6	61.5	56.4
Pyruvic acid	79.0	85.1	74.5	79.1	95.8	95.8
sec-butenyl-acetate	57.5	68.9	63.2	63.7	64.6	73.5
sec-butylbenzene	66.8	69.8	66.3	66.5	65.7	57.4
sec-butyl-formate	63.8	72.0	60.6	61.3	61.7	70.2
succinic-acid	117.6	103.1	110.1	111.9	109.7	124.2
t-butyl-hydroperoxide	63.4	79.4	62.4	67.4	61.0	77.9
tetrachlorothiophene	61.5	70.2	93.0	68.2	67.9	41.0
tetrahydrothiophene	51.0	53.9	57.1	59.0	42.3	85.2
toluene	51.5	56.9	48.4	54.6	51.8	53.6
trans-1,2-dimethylcyclohexane	52.2	51.1	52.0	56.4	53.2	74.5
trans-1,3-hexadiene	46.3	52.6	40.9	43.6	47.2	41.9
trans-2-eicosene	150.3	150.4	142.6	147.1	139.1	131.5
trans-2-pentene	42.3	50.2	40.9	43.6	43.6	53.4
trans-decahydronaphthalene	63.8	55.0	64.4	71.2	59.7	100.9
triacetone alcohol	113.0	95.6	113.7	115.2	97.2	116.6
triallylamine	66.2	76.1	73.2	66.2	77.6	44.0
trichloroacetaldehyde	48.9	73.6	57.4	48.7	56.9	60.0
trichloroacetyl chloride	55.6	71.0	66.0	50.9	58.8	63.1
tridecanal	113.0	106.1	108.1	106.5	109.6	117.4
trimethylamine	35.5	48.9	30.4	37.7	39.5	47.6
undecanal	98.6	92.5	94.6	92.3	97.0	107.0
undecylamine	104.0	95.1	97.9	96.1	104.8	108.8
vinylcyclohexene	52.1	57.3	55.9	59.4	57.2	61.7

Table S2. Comparison of experimental and predicted Gibbs energies of sublimation ($kJ\ mol^{-1}$).

Training set:

	Expt.	Eq. 13	Eq. 14	Eq. 15	Eq. 16
1,1,2-trifluoro-1,2-diphenylethane	64.0	39.9	37.5	39.2	37.9
1,1-di-4-methylphenylethene	49.0	39.3	36.2	38.8	36.2
1,1difluoro-3,3,3-triphenylpropane	76.8	58.2	47.7	51.1	55.3
1,1-dimethylpropylethylpropanedinitrile	23.1	36.7	41.9	34.3	38.3
1,2,3-trihydroxybenzene	53.4	47.7	37.7	44.5	45.2
1,2,4-trichlorobenzene	19.1	18.8	31.9	21.2	27.2
1,2-diacetylhydrazine	49.9	33.8	42.9	34.1	34.3
1,2-dihydroxyanthraquinone	84.6	74.7	63.6	74.5	75.9
1,3-dimethyl-5-fluorouracil	25.8	30.4	38.5	34.5	33.2
1,3-ditertbutyl-5-methylbenzene	24.5	35.7	39.3	29.2	27.4
1,3-ditertbutylUrea	40.7	35.3	41.5	34.4	32.4
1,3-dithiolan-2-thione	32.7	29.2	33.5	39.1	44.4
1,4-bis-2-hydroxyethylpiperazine	66.8	50.0	51.3	47.6	49.2
1,4-bromochlorobenzene	19.5	17.5	26.1	20.4	26.1
1,4-diaminoanthraquinone	81.5	79.0	65.5	72.3	71.6
1,4-diazabicyclo-222-octane	18.6	16.7	23.0	24.3	19.4
1,4-dihydronaphthalene	19.4	29.6	39.9	34.0	33.4
1,4-dimethyl-2,6,7-trioxabicyclo-2,2,2-octane	21.4	21.9	30.9	30.6	23.6
1,4-dimethylbenzene	22.5	15.9	25.7	20.4	19.4
1,4-dithiane	22.9	19.2	28.1	31.8	37.1
1,6-hexanedioic acid	57.5	49.4	49.1	48.8	50.4
1,8-naphthalenediamine	40.6	57.1	49.0	49.2	48.1
1-adamantylmethylketone	27.4	29.2	30.0	35.0	26.9
1-amino-2-bromo-4,4-methylphenylamino-9,10-anthraquinone	120.2	94.3	70.0	88.9	92.9
1-amino-2-methyl-9,10-anthraquinone	93.0	66.9	54.9	65.7	65.5
1-chlorodibenzobe-1,4-dioxin	39.3	62.3	57.8	56.9	56.9
1-dodecanol	34.1	43.4	60.4	44.0	46.9
1H-benzotriazole	43.0	44.3	40.1	42.9	40.3
1-hydroxy-2,2,6,6-tetramethyl-4-oxopipiridine	28.4	36.3	32.0	36.1	33.7
1-nitronaphthalene	36.4	46.4	49.5	50.0	49.6
1N-methylamino-4,3-methylphenylamino-9,10-anthraquinone	103.9	88.9	74.5	86.0	87.2
1-phenyl-3-pyrazolidone	43.4	38.2	37.7	39.6	35.2
2,2,4,4,6,6-hexamethylazobenzene-N,N-dioxide	37.1	59.9	47.9	60.9	60.3
2,2-diaminodiphenylmethane	73.9	55.2	43.9	50.7	47.0
2,2-dihydroxy-4,4-dimethoxybenzop	65.2	67.1	47.5	70.4	70.8
2,2-dihydroxybiphenyl	44.6	47.6	42.3	49.1	48.5
2,2-dimethylbiphenyl	26.0	33.5	32.0	33.4	32.1
2,2-dimethylpropanamide	30.6	26.5	41.7	25.7	24.9
2,2-dinitroadamantane	38.2	45.8	33.7	57.9	50.7
2,2-metacyclophane	37.2	53.6	59.3	54.6	55.6
2,3,5,6-dibenzoxalene	57.6	51.5	55.4	56.9	52.3
2,3,5,6-tetramethylbenzoic acid	46.1	41.2	43.9	38.3	39.3
2,4,5-trichloro-1,1-biphenyl	39.6	36.7	40.5	35.4	39.9
2,4,5-trimethylbenzoic acid	45.1	40.3	42.9	37.7	37.9
2,4,6-triaminostriazine	68.3	75.6	35.5	52.0	47.5
2,4,6-trinitrotoluene	46.3	64.0	41.5	67.9	66.4
2,4,6-tritertbutylaniline	37.3	50.9	52.8	40.7	39.2
2,4-dichloro-1,1-Biphenyl	34.1	33.9	36.5	34.2	36.4
2,4-ditertbutylphenol	30.4	40.1	42.1	36.9	35.8
2,6-dichloro-1,4-benzoquinone	30.5	30.4	38.1	31.1	34.1

2,6-diisopropylnitrobenzene	28.0	34.7	39.0	37.8	40.0
2,6-dimethylphenol	20.7	25.5	33.6	28.8	28.8
2,6-diphenylphenol	51.8	53.1	42.8	55.3	52.9
2,7-dimethylnaphthalene	16.1	34.6	43.1	36.4	35.6
2-acetylaminobenzoic acid	54.4	48.2	40.1	49.0	49.3
2-amino-3-methylpentanoic acid	62.0	62.4	55.5	60.7	62.6
2-amino-6-methylbenzoic acid	47.3	49.1	34.9	43.2	42.9
2-aminoanthraquinone	78.8	69.7	62.4	65.1	63.9
2-azetidinone	23.9	21.7	30.6	23.9	20.2
2-chloro-4,6-bisethylaminostriazine	60.7	46.8	31.3	47.2	49.3
2-chloro-4,6-bisisopropylamino-1,3,5-triazine	57.3	50.4	38.6	50.2	51.0
2-chloro-adamantane	24.2	21.8	20.3	28.1	22.2
2-diphenylmethyl-2-ethyl-1,3-cyclopentanedione	53.3	58.3	51.4	56.8	58.6
2-fluorenyl-2-methyl-1,3-cyclopentandione	64.3	66.4	65.2	66.0	65.3
2-fluoro-1,2,3-triphenylpropane	81.5	56.3	46.5	54.9	52.7
2-fluorobenzoic acid	35.5	34.9	35.8	35.5	35.6
2-furoic acid	30.0	35.5	35.8	36.2	34.8
2-hydroxy1phenylazonaphthalene	85.1	70.2	54.4	73.4	70.9
2-hydroxy4methoxybenzophenone	45.3	51.8	40.6	55.1	54.1
2-hydroxyquinoline	55.7	42.0	50.5	43.8	42.2
2-methyl-3-hydroxyquinoxaline	57.4	45.3	50.3	48.2	46.4
2-methyl-4-hydroxyquinoline	71.7	46.7	54.7	44.6	43.5
2-methyl-pentanedioic acid	49.2	46.7	42.1	46.8	48.9
2-nitro-1,4-dihydroxybenzene	41.2	52.9	30.9	52.1	51.6
2-nitroadamantane	36.1	32.7	29.4	43.4	35.0
2-nitro-benzonitrile	9.5	40.7	34.6	43.5	42.8
2-phenyl-2-methyl-1,3-dioxolane	51.3	29.1	30.8	34.5	30.7
2-phenylacetamide	44.7	38.2	37.5	36.6	34.9
2-phenylacetic acid	35.2	37.7	33.8	37.6	37.0
2-tertbutyl-5-methylphenol	25.4	31.6	34.1	32.9	32.1
2-tertbutylbenzoic acid	37.5	41.8	36.2	39.1	39.1
2-thenoic acid	35.8	35.8	42.4	40.2	41.9
3,4-chlorophenyl-1,1-dimethylurea	55.1	43.2	45.1	38.8	39.8
3,4-dichlorobenzenamine	31.1	33.0	35.0	28.1	31.0
3,5-dimethylphenol	26.1	28.8	35.7	29.1	28.5
3,5-dimethylpyrazole	28.3	23.4	35.9	25.9	23.4
3(5)-trifluoromethyl-5(3)-(methyl)pyrazol	27.2	27.7	34.7	29.6	26.5
3,8-dimethylnaphtho[3,2,1-kl]-xanthene	71.3	70.3	67.6	69.9	69.2
3-acetamidophthalimide	57.0	64.3	55.6	60.5	59.2
3-aminobenzoic acid	55.8	50.8	45.1	42.7	41.5
3-dimethylaminophthalimide	46.0	49.4	52.3	50.8	50.2
3-diphenylmethyl-2,4-pentanedione	48.4	55.7	51.3	52.9	55.5
3-ethoxy-4-hydroxybenzadehyde	38.2	40.6	40.5	42.7	44.0
3-ethylbenzoic acid	36.5	38.8	44.5	38.0	38.6
3-iodobenzoic acid	48.4	39.1	50.2	35.9	42.1
3-methoxy4nitrobenzoic acid	61.5	56.4	44.9	56.7	57.0
3-methyl-2,4,6-trinitrotoluene	57.1	63.8	42.0	68.5	68.0
3-methyl-3-nitro-4-N,N-dimethylamine	54.3	65.1	46.8	70.3	65.5
3-methylaminophthalimide	53.4	58.6	53.0	51.9	51.3
3-methylcholanthrene	68.5	64.3	63.8	65.4	62.0
3-nitroaniline	41.8	45.8	42.8	42.4	40.0
3-nitrophenol	36.7	42.6	41.6	43.4	42.1
3-phenyl-2-propynoic acid	42.1	42.1	48.0	40.5	39.8
3-pyridinecarboxamide	49.0	40.9	41.5	37.2	35.3
4,4-dimethyl-1,3-cyclohexanedione	36.3	27.0	35.3	30.6	27.9
4,5-tetramethylene-1,3-dithiolan-2-thione	46.4	37.5	34.5	47.7	49.8
4,5-tetramethylene-1,3-dithiole-2-thione	43.7	47.2	47.7	56.4	60.9
4-acetomidobenzaldehyde	44.1	43.6	42.9	41.1	39.9

4-amino-N-(2,5-dichlorophenyl)benzenesulfonamide	75.7	72.5	61.7	64.2	70.0
4-benzyloxyphenylacetic acid	57.8	58.2	47.3	62.8	58.1
4-chloroaniline	25.5	30.8	33.1	27.2	27.7
4-heptylbenzoic acid	53.9	54.3	59.6	53.6	53.2
4-hydroxy-4-nitroazobenzene	72.3	77.2	48.3	72.6	66.6
4-methoxy-benzenepropanoic acid	48.4	44.4	46.0	46.7	46.3
4-methylpyridine-N-oxide	37.8	35.9	32.8	31.5	30.4
4-nitro-4-(N,N-dimethyl)aminoazobenzene	72.9	63.3	48.0	70.3	63.9
4-nitrobenzoic acid	53.9	52.1	42.2	51.7	50.2
4-N,N-diethylaminoazobenzene	78.2	52.7	45.8	57.0	54.2
4-N,N-dimethylaminonitrosobenzene	54.4	45.9	38.9	37.9	38.2
4-octyl-benzoic acid	56.4	57.1	64.3	57.1	56.2
4-pentyl-benzoic acid	47.6	48.1	52.3	47.1	47.3
5-alpha-cholestane	61.3	71.2	63.1	62.4	57.8
5-aminoquinoline	43.7	47.3	51.7	45.0	43.7
5-chloro-2-hydroxy-benzophenone	45.0	47.7	43.0	49.9	50.7
5-chloro-7-iodo-8-hydroxy-quinoline	52.2	50.6	53.2	47.8	57.4
5-nitro-8hydroxyquinoline	49.3	59.8	53.4	62.1	62.2
5-phenyl-1,2-dithiole-3-thione	54.7	48.2	41.0	54.4	58.5
5-phenylvaleric acid	42.7	44.5	51.5	46.6	45.5
5-(trifluoromethyl)uracil	32.9	42.8	40.3	40.3	37.4
6-(chloro)uracil	67.8	41.9	39.2	38.6	37.9
7-bromo-5-chloro-8-hydroxyquinoline	49.5	49.7	50.6	47.8	55.2
8,16-pyranthenedione	114.4	111.2	111.6	112.5	113.8
8-aminoquinoline	33.2	47.2	46.8	45.0	43.7
8-butyl-9-methyladenine	63.2	61.4	55.1	60.8	59.9
8-ethyl-9-methyladenine	59.1	58.0	52.0	55.2	53.9
8-nitroquinoline	44.8	49.2	54.0	53.4	52.6
9,10-dimethylanthracene	51.2	49.0	54.6	47.8	48.5
9,9-bifluorenyl	71.3	83.4	83.2	81.0	81.7
9-hydroxy-1,4-anthracenedione	56.6	65.9	69.2	66.0	66.0
9-methyladenine	61.9	56.1	50.0	52.0	49.3
9-methylanthracene	42.2	46.0	52.8	47.4	46.9
acetylsalicylic acid	43.6	44.8	43.8	48.3	48.6
adamantan-2-one	27.2	23.6	24.4	32.6	24.0
adamantyl bromide	25.0	21.9	23.5	27.6	22.1
anthranthrene	78.0	72.7	76.5	74.4	72.3
a-tertbutyl-malononitrile	20.2	30.4	39.5	30.8	31.9
benzanilide	51.3	44.4	44.4	46.8	43.2
benzil	42.3	45.9	41.0	48.3	46.0
benzoapyrene	63.5	65.1	69.2	66.9	65.3
benzobfluorene	57.9	55.5	60.7	58.1	55.0
benzoyltrifluoracetone	24.0	39.3	40.4	42.2	40.5
bicyclo-2,2,2-octane	13.5	12.7	19.1	22.6	17.9
biphenyl	28.5	27.5	30.5	32.0	29.4
bis-2-chloroethylsulfide	21.4	19.6	40.9	25.7	37.4
cis-2-butenoic acid amide	26.3	31.5	40.5	26.6	27.7
cis-2-hexenoic acid amide	28.6	34.9	45.7	31.5	32.8
cis-2-pentenoic acid amide	31.3	32.8	43.6	29.0	30.2
coronene	83.0	85.4	90.6	84.9	84.6
cyanogen dioxide	16.6	23.6	38.4	16.6	26.1
cyclopentadecanone	33.8	43.8	45.8	45.6	47.4
decafluorobiphenyl	25.7	40.3	27.7	41.2	44.3
dibenzo[fg,op]naphthacene	90.3	79.0	82.4	78.0	79.5
dibenzothiophene	38.3	44.9	51.3	51.9	52.3
diformylhydrazine	51.4	38.7	40.7	32.0	33.2
diphenylacetylene	34.2	33.5	36.6	38.8	35.0
diphenylcyclopropenone	55.1	47.7	42.7	50.5	43.8
disperse yellow	94.6	76.6	64.0	80.5	77.4

d,l-2,3-dimethoxy-2,3-diphenylbutane	41.9	52.8	45.3	46.6	49.7
dodecanamide	59.8	48.0	58.2	49.1	49.5
dodecanedioic acid	68.5	68.0	71.3	67.6	67.6
Dodecanoic acid lauric acid	43.0	49.4	67.7	50.1	51.6
flurbiprofen	53.3	54.6	48.5	54.3	53.1
formic acid	5.7	31.1	36.6	22.7	24.5
heneicosanoic acid	66.6	72.2	93.8	83.5	79.2
hexachlorobenzene	44.2	28.0	37.5	23.7	37.7
hexadecanoic acid	55.5	60.0	66.2	64.0	63.6
hexakis(trifluoromethyl)tetracyclo[2.2.0.0(2,6).0(2,5)]hexane	7.8	53.2	46.1	45.3	35.6
hexamethylbenzene	30.6	27.7	30.2	23.7	24.3
homopiperonylic acid	50.4	54.8	55.4	56.8	55.1
LL-proline	64.6	51.7	59.6	58.4	56.6
malononitrile	21.0	23.5	39.2	26.9	28.2
mefenamic acid	59.2	58.8	47.4	56.8	55.8
methyl-butanedioic acid	48.6	46.7	48.6	44.1	46.2
methylcarbamate	22.5	31.5	40.3	26.8	27.3
methylmalonic acid	47.0	45.9	44.7	41.6	43.7
N-acetyl-1-naphthylamine	47.9	47.7	55.9	48.5	48.6
N-acetyl-glycine-N-methylamide	49.4	35.1	46.9	36.3	37.3
N-acetyl-L-isoleucineamide	74.2	45.4	50.7	43.4	45.8
naphthalene	22.4	29.6	40.9	34.0	33.4
N-benzyl-pivalophenone imine	39.4	48.9	46.6	49.0	46.6
N-ethyl-N-methyl Urea	42.5	29.3	40.0	28.1	29.4
niflumic acid	61.3	64.1	46.3	64.2	61.5
N-methyl-N-nitromethanamine	19.0	21.6	32.2	26.8	27.1
N-methyltetradecanamide	49.5	54.4	71.7	55.1	55.4
N-methylthiourea	50.2	66.6	40.7	27.8	30.4
N-methylurea	57.5	33.0	41.9	31.5	33.7
N-nitromorpholine	19.9	29.3	31.8	37.8	35.8
N,N,N,N-tetramethyl-1,5-naphthalenediamine	39.2	47.7	48.7	43.8	46.3
N-[N-(N-[trifluoroacetyl]glycyl)glycyl]glycine methyl ester	60.0	51.9	48.5	57.2	56.4
n-octadecane	39.2	50.8	57.6	53.4	53.5
nonamide	44.2	41.5	56.1	39.8	41.0
N-phenyl-anthranilic acid	58.9	56.9	49.5	54.8	52.9
N-phenylbenzylamine	36.9	39.7	35.9	42.0	37.8
octanamide	42.0	38.7	53.0	36.9	38.2
Oxalic acid monoamide	52.6	65.1	64.1	60.1	60.0
paracetamol	60.0	42.6	44.3	42.1	40.9
PCB126	57.8	42.3	49.0	37.9	47.1
PCB153	51.7	45.9	51.3	38.4	50.9
PCB174	55.1	49.1	55.8	38.5	54.9
PCB22	40.0	36.7	40.7	35.2	40.0
PCB70	47.2	39.7	45.4	36.4	43.5
PCDD26	56.5	64.7	61.5	59.9	64.0
PCDD27	57.5	69.1	63.5	59.7	68.2
PCDD65	68.2	75.6	71.1	61.8	75.8
PCDF2	40.0	45.2	53.7	49.2	49.1
pentacene	96.6	71.6	73.4	75.6	71.3
pentafluorophenol	14.4	29.5	27.2	31.6	32.6
pentamethoxycarbonylbenzene	73.3	81.5	75.0	82.3	94.3
pentamethylbenzene	26.2	24.7	28.7	22.9	23.0
perylene	72.7	65.1	69.0	65.7	65.4
phenazine	23.8	46.8	53.2	53.8	51.3
phenothiazine	49.5	61.5	56.7	61.7	61.5
phenylsalicylate	36.1	48.5	38.1	54.7	52.2
picene	83.2	70.5	73.7	73.4	71.9

proline, 1-[N-(trifluoroacetyl)-1-leucyl] methyl ester	50.6	64.8	61.8	67.0	69.1
pterphenyl	56.8	43.5	38.7	48.5	43.0
ptoluidine	20.7	30.2	35.1	27.2	25.4
pyracene	46.5	50.8	58.2	52.9	51.7
resorcinol-di-benzoate	69.2	68.0	49.0	77.9	70.9
sesamol	32.0	43.8	47.4	45.8	44.2
succinimide	36.7	30.3	33.1	30.5	27.5
tetradecanamide	64.1	57.7	76.9	55.9	55.4
tetrahydro-4H-thiopyran-4-one	20.1	21.3	29.3	30.5	32.2
tetraphenylmethane	71.5	65.5	51.1	57.2	61.2
thiocamphor	25.3	28.8	30.3	31.5	27.0
trans-2-nonenoic acid amide	44.3	42.9	53.8	39.8	41.0
trans-2-octenoic acid amide	33.0	50.3	52.0	51.6	55.5
trans-3,5-dimethoxycinnamic acid	62.9	53.5	46.7	51.9	53.0
trans-3-phenyl-2-propen-1-ol	31.4	33.1	39.0	34.6	35.2
trichloro-1,4-benzoquinone	36.4	32.6	41.2	31.9	37.6
trifluoroacetamide	19.8	27.7	40.2	25.7	24.9
triphenylamine	41.8	46.6	42.0	48.6	47.3
triphenylene	65.2	57.1	63.1	58.1	58.6
Violanthrene-B	103.4	116.0	112.4	117.3	117.1

Test set:

	Expt.	Eq. 13	Eq. 14	Eq. 15	Eq. 16
1,3,5-trimethyluracil	41.4	29.2	33.8	34.9	33.9
1,3-dimethyl-5-butyluracil	40.7	55.0	37.4	51.2	55.3
1,3-dimethyl-5-propyluracil	25.8	34.3	36.4	39.4	40.0
1-naphthylamine	33.0	44.2	45.6	41.9	41.2
2,2-dicyanopropionitrile	21.0	32.9	29.4	36.2	37.7
2,3dimethylnaphthalene	30.6	34.8	38.3	36.4	36.1
2,4,6-trimethylphenol	27.9	30.3	29.2	27.7	26.9
2,5-thiophenedicarboxylic acid	72.7	58.5	40.0	59.0	60.5
2-aminobenzoic acid	42.8	49.5	30.0	43.5	42.9
2-methoxybenzoic acid	43.7	38.5	32.6	41.0	42.0
3-benzalphthalide	57.7	53.5	47.1	57.9	54.6
3-tert-butylphenol	27.7	35.3	32.3	29.6	27.9
4-hexylbenzoic acid	50.5	51.4	39.8	51.3	51.1
4-phenylbutyric acid	39.0	40.6	32.9	44.5	43.6
5,7-dihydro-6H-dibenzo(a,c) cyclohepten-6-one	70.3	53.4	50.6	53.6	54.2
5-methyl-2-nitrobenzoic acid	51.6	59.8	32.0	54.0	53.8
9-butylanthracene	45.6	56.1	52.9	54.6	56.9
acetate-1-Naphthalenol	34.1	43.2	42.1	48.1	48.3
acridine	41.0	45.0	49.3	50.6	48.7
benzene	0.9	10.1	19.8	18.7	17.8
Butylparaben	42.2	46.8	37.9	47.1	46.4
d-3-bornanone	20.0	26.4	24.5	28.0	22.0
di-tertbutylmethanol	16.3	30.2	30.1	23.7	23.1
fluorene	34.7	40.9	45.4	44.7	42.9
heptanamide	37.9	38.2	38.3	34.5	35.9
Methylparaben	42.2	39.6	35.6	38.6	37.8
N,N-tri-fluoroacetyl-valyl-alanine-Ethyl ester	50.5	62.8	46.2	64.7	67.8
N-tertbutylurea	59.8	41.7	31.5	31.7	30.7
PCB25	39.6	35.7	27.1	35.5	40.2
PCDF43	48.6	51.1	51.2	51.2	56.8
ppDDT	52.9	49.4	32.1	42.0	51.0
salicylic acid	38.5	47.8	35.0	41.9	41.5
tri-chloro-hydroquinone	40.4	47.2	29.7	33.9	39.7
tropolone	25.6	32.6	29.2	32.0	31.4

Table S3. Comparison of experimental and predicted Gibbs energies of sublimation from neural network modelling (kJ mol^{-1}).

Training set:

	Expt.	Eq. 13	Eq. 14	Eq. 15	Eq. 16
1,1,2-trifluoro-1,2-diphenylethane	64.0	42.4	41.0	40.9	41.9
1,1-di-4-methylphenylethene	49.0	40.5	41.3	40.5	39.5
1,1difluoro-3,3,3-triphenylpropane	76.8	64.2	49.8	54.6	59.7
1,1-dimethylpropylethylpropanedinitrile	23.1	29.1	46.1	36.0	40.9
1,2,3-trihydroxybenzene	53.4	48.0	33.4	39.4	39.8
1,2,4-trichlorobenzene	19.1	20.4	32.8	18.5	26.0
1,2-diacetylhydrazine	49.9	47.3	42.0	36.8	38.5
1,2-dihydroxyanthraquinone	84.6	73.2	67.0	75.2	78.3
1,3-dimethyl-5-fluorouracil	25.8	30.2	35.5	36.0	36.1
1,3-ditertbutyl-5-methylbenzene	24.5	31.9	44.9	28.8	28.6
1,3-ditertbutylUrea	40.7	39.5	45.6	35.7	34.7
1,3-dithiolan-2-thione	32.7	25.5	28.8	43.0	45.8
1,4-bis-2-hydroxyethylpiperazine	66.8	60.7	55.5	43.3	43.2
1,4-bromochlorobenzene	19.5	17.8	21.2	17.4	24.1
1,4-diaminoanthraquinone	81.5	78.6	70.5	78.8	81.6
1,4-diazabicyclo-222-octane	18.6	17.6	14.9	22.8	14.7
1,4-dihydronaphthalene	19.4	17.6	27.4	20.7	24.2
1,4-dimethyl-2,6,7-trioxabicyclo-2,2,2-octane	21.4	24.8	25.5	31.1	22.5
1,4-dimethylbenzene	22.5	18.4	20.6	17.4	14.5
1,4-dithiane	22.9	22.9	23.2	34.0	39.4
1,6-hexanedioic acid	57.5	47.2	52.7	54.2	53.2
1,8-naphthalenediamine	40.6	49.1	41.4	46.5	46.5
1-adamantylmethylketone	27.4	29.2	27.9	35.6	26.5
1-amino-2-bromo-4,4-methylphenylamino-9,10-anthraquinone	120.2	100.1	86.1	108.6	111.2
1-amino-2-methyl-9,10-anthraquinone	93.0	72.1	51.7	69.7	72.2
1-chlorodibenzobe-1,4-dioxin	39.3	42.3	53.8	54.8	53.3
1-dodecanol	34.1	42.4	53.6	42.4	38.1
1H-benzotriazole	43.0	42.8	26.1	35.6	35.3
1-hydroxy-2,2,6,6-tetramethyl-4-oxopipiridine	28.4	36.2	33.2	34.9	32.7
1-nitronaphthalene	36.4	46.1	43.7	48.1	46.9
1N-methylamino-4,3-methylphenylamino-9,10-anthraquinone	103.9	95.4	92.7	106.1	102.2
1-phenyl-3-pyrazolidone	43.4	40.6	36.5	40.4	35.7
2,2,4,4,6,6-hexamethylazobenzene-N,N-dioxide	37.1	51.2	50.3	57.7	63.8
2,2-diaminodiphenylmethane	73.9	64.2	46.9	49.9	49.2
2,2-dihydroxy-4,4-dimethoxybenzop	65.2	65.9	50.8	59.9	64.0
2,2-dihydroxybiphenyl	44.6	56.7	44.0	44.5	43.0
2,2-dimethylbiphenyl	26.0	36.1	33.6	34.0	34.1
2,2-dimethylpropanamide	30.6	28.1	39.5	27.2	26.7
2,2-dinitroadamantane	38.2	36.6	35.4	55.0	54.6
2,2-metacyclophane	37.2	51.2	52.2	48.5	50.9
2,3,5,6-dibenzoxalene	57.6	50.8	49.6	57.9	48.3
2,3,5,6-tetramethylbenzoic acid	46.1	39.2	46.7	39.6	40.1
2,4,5-trichloro-1,1-biphenyl	39.6	35.2	45.7	36.5	42.8
2,4,5-trimethylbenzoic acid	45.1	38.5	44.6	39.1	38.3
2,4,6-triaminostriazine	68.3	67.1	29.5	53.6	45.4
2,4,6-trinitrotoluene	46.3	54.6	45.6	61.6	54.2
2,4,6-tritertbutylaniline	37.3	39.8	51.9	41.5	41.7
2,4-dichloro-1,1-Biphenyl	34.1	34.4	40.8	35.0	38.8
2,4-ditertbutylphenol	30.4	36.8	46.8	35.0	32.6
2,6-dichloro-1,4-benzoquinone	30.5	27.3	38.1	32.0	34.5
2,6-diisopropylnitrobenzene	28.0	35.3	42.5	38.6	40.1
2,6-dimethylphenol	20.7	22.2	29.2	25.9	22.7

2,6-diphenylphenol	51.8	60.8	47.4	53.7	50.2
2,7-dimethylnaphthalene	16.1	28.1	33.5	25.2	28.2
2-acetylaminobenzoic acid	54.4	49.7	42.1	51.9	52.4
2-amino-3-methylpentanoic acid	62.0	60.2	52.1	60.5	63.0
2-amino-6-methylbenzoic acid	47.3	50.2	33.2	47.4	46.6
2-aminoanthraquinone	78.8	77.0	63.7	68.4	69.2
2-azetidinone	23.9	15.2	20.8	23.9	17.3
2-chloro-4,6-bisethylaminostriazine	60.7	58.7	37.6	47.3	50.0
2-chloro-4,6-bisisopropylamino-1,3,5-triazine	57.3	60.1	44.0	49.5	52.3
2-chloro-adamantane	24.2	25.6	16.3	27.4	22.0
2-diphenylmethyl-2-ethyl-1,3-cyclopentanedione	53.3	54.1	51.9	56.1	59.3
2-fluorenyl-2-methyl-1,3-cyclopentandione	64.3	69.0	75.8	73.5	68.7
2-fluoro-1,2,3-triphenylpropane	81.5	62.2	49.1	58.8	57.3
2-fluorobenzoic acid	35.5	32.4	32.0	37.1	35.1
2-furoic acid	30.0	30.9	30.5	39.7	36.1
2-hydroxy1phenylazonaphthalene	85.1	72.5	60.2	80.9	68.0
2-hydroxy4methoxybenzophenone	45.3	50.5	45.4	51.6	51.5
2-hydroxyquinoline	55.7	42.5	42.6	37.6	36.0
2-methyl-3-hydroxyquinoxaline	57.4	46.7	43.3	44.9	43.6
2-methyl-4-hydroxyquinoline	71.7	40.4	51.2	38.9	37.8
2-methyl-pentanedioic acid	49.2	43.3	44.0	52.5	52.0
2-nitro-1,4-dihydroxybenzene	41.2	50.9	28.7	48.5	47.1
2-nitroadamantane	36.1	30.0	26.0	44.0	39.4
2-nitro-benzonitrile	9.5	37.6	33.4	45.0	46.4
2-phenyl-2-methyl-1,3-dioxolane	51.3	31.0	29.7	35.1	29.9
2-phenylacetamide	44.7	40.6	36.3	37.9	36.9
2-phenylacetic acid	35.2	37.6	32.8	39.0	36.6
2-tertbutyl-5-methylphenol	25.4	33.4	35.7	30.5	27.3
2-tertbutylbenzoic acid	37.5	43.0	38.9	40.4	39.9
2-thenoic acid	35.8	32.1	40.1	45.4	44.9
3,4-chlorophenyl-1,1-dimethylurea	55.1	40.2	48.9	39.6	39.2
3,4-dichlorobenzenamine	31.1	32.5	33.9	28.3	29.9
3,5-dimethylphenol	26.1	28.5	32.1	26.3	22.3
3,5-dimethylpyrazole	28.3	22.7	28.7	26.1	21.7
3(5)-trifluoromethyl-5(3)-(methyl)pyrazol	27.2	27.4	28.8	30.2	25.7
3,8-dimethylnaphtho[3,2,1-kl]-xanthene	71.3	75.3	72.9	77.3	73.4
3-acetamidophthalimide	57.0	68.3	55.5	63.6	60.7
3-aminobenzoic acid	55.8	49.7	45.2	47.0	45.0
3-dimethylaminophthalimide	46.0	40.4	51.7	49.2	49.1
3-diphenylmethyl-2,4-pentanedione	48.4	53.3	53.4	52.6	54.5
3-ethoxy-4-hydroxybenzadehyde	38.2	39.8	42.9	41.2	42.2
3-ethylbenzoic acid	36.5	36.9	46.4	39.4	38.5
3-iodobenzoic acid	48.4	36.6	52.0	37.4	41.7
3-methoxy4nitrobenzoic acid	61.5	55.5	48.1	59.2	56.8
3-methyl-2,4,6-trinitrotoluene	57.1	53.0	46.5	61.8	55.6
3-methyl-3-nitro-4-N,N-dimethylamine	54.3	61.2	50.6	63.5	65.5
3-methylaminophthalimide	53.4	56.0	48.5	50.8	51.1
3-methylcholanthrene	68.5	67.6	62.4	69.3	62.2
3-nitroaniline	41.8	46.5	41.7	44.4	44.4
3-nitrophenol	36.7	41.3	40.1	42.8	43.3
3-phenyl-2-propynoic acid	42.1	43.6	51.5	41.6	39.6
3-pyridinecarboxamide	49.0	42.4	38.7	39.2	39.5
4,4-dimethyl-1,3-cyclohexanedione	36.3	26.8	33.2	31.5	28.5
4,5-tetramethylene-1,3-dithiolan-2-thione	46.4	29.0	34.4	48.6	50.0
4,5-tetramethylene-1,3-dithiole-2-thione	43.7	36.7	40.9	57.0	56.6
4-acetomidobenzaldehyde	44.1	46.3	44.7	42.0	41.7
4-amino-N-(2,5-dichlorophenyl) benzenesulfonamide	75.7	72.6	63.1	58.8	64.2
4-benzyloxyphenylacetic acid	57.8	61.5	51.3	58.5	58.5

4-chloroaniline	25.5	28.0	29.5	27.4	26.2
4-heptylbenzoic acid	53.9	50.0	58.0	51.9	52.2
4-hydroxy-4-nitroazobenzene	72.3	82.2	52.3	62.3	60.1
4-methoxy-benzenepropanoic acid	48.4	43.7	49.3	47.9	47.3
4-methylpyridine-N-oxide	37.8	30.8	27.2	29.7	26.8
4-nitro-4-(N,N-dimethyl)aminoazobenzene	72.9	62.2	51.0	63.5	63.5
4-nitrobenzoic acid	53.9	51.7	43.3	55.5	52.5
4-N,N-diethylaminoazobenzene	78.2	50.7	49.8	57.0	51.7
4-N,N-dimethylaminonitrosobenzene	54.4	55.3	38.9	36.4	35.2
4-octyl-benzoic acid	56.4	51.0	58.8	54.3	54.6
4-pentyl-benzoic acid	47.6	45.3	55.6	47.1	47.1
5-alpha-cholestane	61.3	57.6	46.6	67.1	63.0
5-aminoquinoline	43.7	41.2	44.8	39.5	38.9
5-chloro-2-hydroxy-benzophenone	45.0	44.8	47.5	47.5	47.2
5-chloro-7-iodo-8-hydroxy-quinoline	52.2	44.5	50.4	40.3	47.8
5-nitro-8hydroxyquinoline	49.3	63.3	50.8	62.1	59.3
5-phenyl-1,2-dithiole-3-thione	54.7	41.4	43.7	52.9	53.6
5-phenylvaleric acid	42.7	43.4	55.4	46.7	45.1
5-(trifluoromethyl)uracil	32.9	48.7	37.3	41.9	42.5
6-(chloro)uracil	67.8	45.5	35.9	41.1	42.7
7-bromo-5-chloro-8-hydroxyquinoline	49.5	43.1	46.0	40.3	46.3
8,16-pyranthenedione	114.4	111.7	108.7	108.8	113.3
8-aminoquinoline	33.2	40.9	36.9	39.5	38.9
8-butyl-9-methyladenine	63.2	67.0	56.6	64.4	60.5
8-ethyl-9-methyladenine	59.1	59.8	47.7	55.8	54.6
8-nitroquinoline	44.8	51.1	50.7	53.2	52.0
9,10-dimethylanthracene	51.2	46.5	49.5	40.2	43.3
9,9-bifluorenyl	71.3	93.0	100.9	90.3	91.7
9-hydroxy-1,4-anthracenedione	56.6	69.8	76.5	65.9	66.3
9-methyladenine	61.9	50.8	41.1	50.6	49.5
9-methylanthracene	42.2	41.5	45.1	39.3	40.8
Acetylsalicylic acid	43.6	41.5	45.7	51.1	51.7
adamantan-2-one	27.2	26.0	17.8	32.9	22.3
adamantylbromide	25.0	25.7	17.3	26.7	22.4
anthranthrene	78.0	74.1	73.3	76.8	79.8
a-tertbutyl-malononitrile	20.2	26.6	40.9	32.7	35.0
benzanilide	51.3	48.9	48.2	47.2	42.5
benzil	42.3	46.1	45.3	48.4	45.7
benzoapyrene	63.5	66.2	65.6	67.2	67.9
benzobfluorene	57.9	53.9	54.7	55.1	50.8
benzoyltrifluoracetone	24.0	33.9	42.4	42.8	41.2
bicyclo-2,2,2-octane	13.5	19.4	13.0	20.3	13.1
biphenyl	28.5	29.5	30.4	32.3	29.9
bis-2-chloroethylsulfide	21.4	23.3	44.8	25.6	32.7
cis-2-butenoic acid amide	26.3	28.6	37.7	28.2	29.6
cis-2-hexenoic acid amide	28.6	35.6	48.2	33.1	34.5
cis-2-pentenoic acid amide	31.3	32.2	43.8	30.6	32.1
coronene	83.0	79.2	83.2	90.2	86.6
cyanogendioxide	16.6	2.6	29.7	14.6	22.9
cyclopentadecanone	33.8	37.6	49.6	46.8	43.4
decafluorobiphenyl	25.7	34.1	28.5	43.3	50.3
dibenzo[fg,op]naphthacene	90.3	83.9	84.0	81.1	90.0
dibenzothiophene	38.3	40.8	41.5	45.2	46.1
diformylhydrazine	51.4	51.0	37.3	34.9	37.3
diphenylacetylene	34.2	36.1	41.1	40.5	36.7
diphenylcyclopropenone	55.1	52.4	46.4	49.0	41.5
disperseyellow	94.6	84.4	76.0	87.8	77.4
d,l-2,3-dimethoxy-2,3-diphenylbutane	41.9	60.1	48.6	47.7	51.1
dodecanamide	59.8	49.0	58.7	48.8	45.9
dodecanedioic acid	68.5	67.3	61.2	66.6	67.1

dodecanoic acid lauric acid	43.0	42.7	60.2	49.4	48.7
flurbiprofen	53.3	54.2	52.5	52.4	54.6
formic acid	5.7	3.8	29.2	20.5	20.0
heneicosanoic acid	66.6	63.7	65.1	72.1	66.8
hexachlorobenzene	44.2	28.8	43.7	21.8	38.5
hexadecenoic acid	55.5	55.5	55.7	58.9	57.2
hexakis(trifluoromethyl)tetracyclo [2.2.0.0(2,6).0(2,5)]hexane	7.8	34.8	46.6	48.0	8.6
hexamethylbenzene	30.6	29.7	31.5	21.8	23.3
homopiperonylic acid	50.4	55.5	54.5	45.7	52.2
LL-proline	64.6	62.8	57.4	61.2	59.8
malononitrile	21.0	23.7	35.8	29.0	31.1
mefenamic acid	59.2	63.1	51.5	55.0	58.1
methyl-butanedioic acid	48.6	43.9	50.6	50.0	49.1
methylcarbamate	22.5	27.1	36.1	29.4	31.0
methylmalonic acid	47.0	42.6	44.3	47.6	46.1
N-acetyl-1-naphthylamine	47.9	46.0	57.0	46.4	43.1
N-acetyl-glycine-N-methylamide	49.4	48.9	48.3	38.6	40.9
N-acetyl-L-isoleucineamide	74.2	51.2	55.0	45.1	48.0
naphthalene	22.4	15.6	28.3	20.7	24.2
N-benzyl-pivalophenoneimine	39.4	48.0	49.3	50.7	48.2
N-ethyl-N-methyl Urea	42.5	29.2	37.2	30.1	32.1
Niflumic acid	61.3	67.7	50.4	60.2	65.4
N-methyl-N-nitromethanamine	19.0	21.6	25.6	29.1	30.3
N-methyltetradecanamide	49.5	50.1	57.8	55.1	47.5
N-methylthiourea	50.2	51.2	36.4	30.8	34.7
N-methylurea	57.5	51.8	36.8	35.8	38.5
N-nitromorpholine	19.9	28.5	26.6	39.9	40.3
N,N,N,N-tetramethyl-1,5-naphthalenediamine	39.2	47.0	48.5	39.0	42.2
N-[N-(N-[trifluoroacetyl]glycyl)glycyl] glycinemethylester	60.0	53.4	52.6	54.4	54.9
n-octadecane	39.2	42.0	46.6	57.1	49.7
nonamide	44.2	41.4	58.6	40.8	40.6
N-phenyl-anthranilic acid	58.9	62.0	53.5	53.8	54.7
N-phenylbenzylamine	36.9	44.2	37.7	43.3	37.3
octanamide	42.0	39.4	56.9	38.2	38.7
oxalic acid monoamide	52.6	52.6	63.5	57.0	56.8
paracetamol	60.0	49.2	45.3	40.8	40.4
PCB126	57.8	34.8	49.9	39.4	50.1
PCB153	51.7	35.9	49.6	40.0	53.5
PCB174	55.1	35.9	50.3	40.1	56.7
PCB22	40.0	35.2	45.6	36.2	42.9
PCB70	47.2	35.6	49.0	37.6	46.6
PCDD26	56.5	58.3	67.9	61.2	61.8
PCDD27	57.5	59.8	73.3	60.8	66.3
PCDD65	68.2	72.3	87.7	65.0	74.2
PCDF2	40.0	38.6	46.7	41.4	42.4
pentacene	96.6	76.9	75.3	81.4	77.4
pentafluorophenol	14.4	29.7	19.8	29.1	27.9
pentamethoxycarbonylbenzene	73.3	76.2	56.5	67.9	76.6
pentamethylbenzene	26.2	27.6	28.0	20.7	21.0
perylene	72.7	66.2	65.3	65.5	68.2
phenazine	23.8	42.9	44.3	49.4	45.1
phenothiazine	49.5	43.3	51.2	62.2	61.7
phenylsalicylate	36.1	48.3	41.8	51.2	49.6
picene	83.2	74.2	75.6	77.9	78.1
proline,1-[N-(trifluoroacetyl)-1-leucyl] methyl ester	50.6	49.5	57.5	61.0	69.4
pterphenyl	56.8	45.8	44.0	51.7	47.3
ptoluidine	20.7	27.9	30.2	27.4	23.6

pyracene	46.5	48.2	57.9	48.2	48.1
resorcinol-di-benzoate	69.2	66.5	51.7	72.5	69.0
sesamol	32.0	34.4	36.9	36.4	35.3
succinimide	36.7	27.7	25.3	32.4	30.6
tetradecanamide	64.1	55.7	61.4	54.5	49.3
tetrahydro-4H-thiopyran-4-one	20.1	23.7	23.7	32.0	34.2
tetraphenylmethane	71.5	78.9	49.9	61.4	65.0
thiocamphor	25.3	27.5	28.7	32.4	27.9
trans-2-nonenoic acid amide	44.3	44.5	57.1	40.8	40.6
trans-2-octenoic acid amide	33.0	50.6	54.9	50.5	51.6
trans-3,5-dimethoxycinnamic acid	62.9	53.2	50.8	52.5	54.5
trans-3-phenyl-2-propen-1-ol	31.4	33.3	40.7	32.4	29.3
trichloro-1,4-benzoquinone	36.4	27.3	43.7	32.9	37.6
trifluoroacetamide	19.8	26.4	34.1	27.2	26.7
triphenylamine	41.8	48.5	46.9	51.4	50.6
triphenylene	65.2	56.5	59.9	55.1	56.1
Violanthrene-B	103.4	109.3	101.5	104.9	98.7

Test set:

	Expt.	Eq. 13	Eq. 14	Eq. 15	Eq. 16
1,3,5-trimethyluracil	41.4	30.5	24.9	36.0	36.1
1,3-dimethyl-5-butyluracil	40.7	64.4	33.6	54.6	59.7
1,3-dimethyl-5-propyluracil	25.8	34.0	31.0	40.1	41.5
1-naphthylamine	33.0	36.9	32.7	33.8	33.3
2,2-dicyanopropionitrile	21.0	26.4	17.7	39.4	41.5
2,3dimethylnaphthalene	30.6	28.2	24.2	24.7	28.4
2,4,6-trimethylphenol	27.9	30.5	19.7	27.2	24.1
2,5-thiophenedicarboxylic acid	72.7	60.6	31.6	63.2	57.9
2-aminobenzoic acid	42.8	50.0	19.4	46.8	45.1
2-methoxybenzoic acid	43.7	39.6	23.1	42.4	42.2
3-benzalphthalide	57.7	58.3	37.1	63.2	50.7
3-tert-butylphenol	27.7	36.4	24.5	29.4	25.6
4-hexylbenzoic acid	50.5	48.3	39.0	49.5	49.7
4-phenylbutyric acid	39.0	41.3	25.5	44.2	42.4
5,7-dihydro-6H-dibenzo(a,c) cyclohepten-6-one	70.3	57.1	39.3	49.4	48.6
5-methyl-2-nitrobenzoic acid	51.6	62.9	23.8	56.6	54.3
9-butylanthracene	45.6	54.8	45.2	53.9	53.9
acetate-1-Naphthalenol	34.1	41.0	29.1	45.2	42.2
acridine	41.0	40.3	36.9	43.9	41.0
benzene	0.9	3.4	9.0	14.7	10.8
Butylparaben	42.2	47.3	33.8	46.8	46.1
d-3-bornanone	20.0	27.0	16.4	27.4	17.7
di-tertbutylmethanol	16.3	30.8	23.2	22.5	19.0
fluorene	34.7	31.4	31.6	33.5	34.0
heptanamide	37.9	37.3	31.4	35.6	36.6
Methylparaben	42.2	41.9	26.5	39.5	39.7
N,N-tri-fluoroacetyl-valyl-alanine-ethyl ester	50.5	54.6	50.0	58.8	64.2
N-tertbutylurea	59.8	47.4	21.2	34.0	34.6
PCB25	39.6	35.2	22.4	36.3	42.8
PCDF43	48.6	41.0	40.2	45.1	52.7
ppDDT	52.9	39.7	33.9	44.0	54.3
salicylic acid	38.5	47.9	24.7	44.7	43.2
tri-chloro-hydroquinone	40.4	55.6	19.9	34.9	40.1
tropolone	25.6	31.7	17.8	32.8	31.9

Table S4. Comparison of experimental and predicted Gibbs energies of sublimation from support vector regression modelling (kJ mol^{-1}).

Training set:

	Expt.	Eq. 13	Eq. 14	Eq. 15	Eq. 16
1,1,2-trifluoro-1,2-diphenylethane	64.0	39.3	37.6	38.6	40.6
1,1-di-4-methylphenylethene	49.0	39.5	36.4	38.2	38.1
1,1difluoro-3,3,3-triphenylpropane	76.8	54.4	47.1	51.1	58.2
1,1-dimethylpropylethylpropanedinitrile	23.1	34.1	41.8	33.5	39.1
1,2,3-trihydroxybenzene	53.4	57.5	38.1	37.0	37.5
1,2,4-trichlorobenzene	19.1	20.4	32.3	20.3	23.1
1,2-diacetylhydrazine	49.9	44.7	43.1	34.1	34.9
1,2-dihydroxyanthraquinone	84.6	77.9	60.9	75.7	77.6
1,3-dimethyl-5-fluorouracil	25.8	28.0	38.9	33.4	35.2
1,3-ditertbutyl-5-methylbenzene	24.5	36.6	39.4	28.0	31.2
1,3-ditertbutylUrea	40.7	44.8	41.4	33.1	34.5
1,3-dithiolan-2-thione	32.7	28.1	34.0	39.4	42.9
1,4-bis-2-hydroxyethylpiperazine	66.8	55.3	50.7	41.9	41.2
1,4-bromochlorobenzene	19.5	19.3	26.9	19.5	21.1
1,4-diaminoanthraquinone	81.5	81.6	62.6	78.9	81.0
1,4-diazabicyclo-222-octane	18.6	18.5	24.0	22.7	18.7
1,4-dihydronaphthalene	19.4	23.4	38.9	25.6	22.1
1,4-dimethyl-2,6,7-trioxabicyclo-2,2,2-octane	21.4	22.0	31.5	28.6	26.8
1,4-dimethylbenzene	22.5	19.4	26.5	19.5	17.2
1,4-dithiane	22.9	18.7	28.8	31.4	33.5
1,6-hexanedioic acid	57.5	51.2	48.8	53.2	52.6
1,8-naphthalenediamine	40.6	55.3	47.5	46.0	44.3
1-adamantylmethylketone	27.4	29.1	30.5	32.9	31.0
1-amino-2-bromo-4,4-methylphenylamino-9,10-anthraquinone	120.2	107.3	66.9	103.1	105.9
1-amino-2-methyl-9,10-anthraquinone	93.0	70.2	52.8	70.0	72.4
1-chlorodibenzobe-1,4-dioxin	39.3	44.5	55.5	55.2	52.7
1-dodecanol	34.1	44.0	58.7	39.7	36.9
1H-benzotriazole	43.0	33.4	39.1	36.6	31.8
1-hydroxy-2,2,6,6-tetramethyl-4-oxopipiridine	28.4	36.4	32.4	32.3	34.2
1-nitronaphthalene	36.4	41.1	48.0	46.9	45.5
1N-methylamino-4,3-methylphenylamino-9,10-anthraquinone	103.9	102.6	71.0	98.9	100.3
1-phenyl-3-pyrazolidone	43.4	38.5	38.0	38.0	35.4
2,2,4,4,6,6-hexamethylazobenzene-N,N-dioxide	37.1	55.0	47.3	61.6	62.6
2,2-diaminodiphenylmethane	73.9	58.3	43.7	50.7	48.5
2,2-dihydroxy-4,4-dimethoxybenzop	65.2	69.0	47.0	66.7	64.7
2,2-dihydroxybiphenyl	44.6	49.8	42.3	43.0	42.6
2,2-dimethylbiphenyl	26.0	34.3	32.4	32.4	32.9
2,2-dimethylpropanamide	30.6	28.2	41.9	25.4	26.6
2,2-dinitroadamantane	38.2	43.7	34.0	58.0	56.5
2,2-metacyclophane	37.2	53.3	56.6	51.2	46.8
2,3,5,6-dibenzoxalene	57.6	48.5	53.2	56.2	51.7
2,3,5,6-tetramethylbenzoic acid	46.1	40.3	43.8	39.8	42.7
2,4,5-trichloro-1,1-biphenyl	39.6	35.7	40.4	34.5	39.8
2,4,5-trimethylbenzoic acid	45.1	39.2	42.9	39.3	40.3
2,4,6-triaminostriazine	68.3	68.2	35.9	47.2	47.1
2,4,6-trinitrotoluene	46.3	55.5	41.3	56.5	55.5
2,4,6-tritertbutylaniline	37.3	49.2	51.8	38.9	44.2
2,4-dichloro-1,1-Biphenyl	34.1	33.5	36.7	33.2	35.9
2,4-ditertbutylphenol	30.4	39.8	41.9	32.3	35.1
2,6-dichloro-1,4-benzoquinone	30.5	27.3	38.3	29.5	32.8
2,6-diisopropylnitrobenzene	28.0	35.7	39.0	36.0	40.5

2,6-dimethylphenol	20.7	24.8	34.1	24.3	24.1
2,6-diphenylphenol	51.8	51.8	42.6	51.6	50.8
2,7-dimethylnaphthalene	16.1	30.2	41.9	28.9	27.5
2-acetylaminobenzoic acid	54.4	53.0	40.1	51.9	53.1
2-amino-3-methylpentanoic acid	62.0	62.2	56.9	61.9	62.0
2-amino-6-methylbenzoic acid	47.3	50.8	35.2	46.1	46.7
2-aminoanthraquinone	78.8	71.0	59.7	68.9	68.8
2-azetidinone	23.9	23.7	31.3	22.7	19.7
2-chloro-4,6-bisethylaminostriazine	60.7	53.1	32.0	47.5	49.0
2-chloro-4,6-bisisopropylamino-1,3,5-triazine	57.3	56.6	38.6	50.7	51.9
2-chloro-adamantane	24.2	24.4	21.7	26.9	24.9
2-diphenylmethyl-2-ethyl-1,3-cyclopentanedione	53.3	52.6	50.5	56.0	59.8
2-fluorenyl-2-methyl-1,3-cyclopentandione	64.3	69.7	62.5	69.9	74.4
2-fluoro-1,2,3-triphenylpropane	81.5	53.6	46.0	54.8	55.8
2-fluorobenzoic acid	35.5	33.1	36.2	37.4	35.6
2-furoic acid	30.0	32.7	36.3	38.9	34.7
2-hydroxy1phenylazonaphthalene	85.1	76.9	52.6	77.5	71.5
2-hydroxy4methoxybenzophenone	45.3	50.5	40.5	52.4	51.7
2-hydroxyquinoline	55.7	34.0	48.9	37.6	34.2
2-methyl-3-hydroxyquinoxaline	57.4	38.5	48.8	44.3	41.8
2-methyl-4-hydroxyquinoline	71.7	35.2	53.0	38.7	36.9
2-methyl-pentanedioic acid	49.2	49.4	42.1	51.4	52.5
2-nitro-1,4-dihydroxybenzene	41.2	52.1	31.4	47.1	47.7
2-nitroadamantane	36.1	31.0	30.0	42.7	39.8
2-nitro-benzonitrile	9.5	39.0	35.0	43.8	44.8
2-phenyl-2-methyl-1,3-dioxolane	51.3	28.9	31.3	32.4	30.9
2-phenylacetamide	44.7	38.1	37.8	35.5	34.4
2-phenylacetic acid	35.2	35.6	34.2	39.2	36.0
2-tertbutyl-5-methylphenol	25.4	31.6	34.4	28.2	29.4
2-tertbutylbenzoic acid	37.5	40.9	36.4	40.5	42.5
2-thenoic acid	35.8	34.0	42.7	43.3	41.4
3,4-chlorophenyl-1,1-dimethylurea	55.1	37.4	44.8	37.1	38.5
3,4-dichlorobenzenamine	31.1	31.6	35.3	26.2	28.4
3,5-dimethylphenol	26.1	27.4	36.1	24.6	23.9
3,5-dimethylpyrazole	28.3	21.8	36.5	24.3	22.8
3(5)-trifluoromethyl-5(3)-(methyl)pyrazol	27.2	25.2	35.2	27.7	27.6
3,8-dimethylnaphtho[3,2,1-kl]-xanthene	71.3	75.4	64.4	75.1	78.6
3-acetamidophthalimide	57.0	66.9	53.7	62.6	61.9
3-aminobenzoic acid	55.8	50.7	45.2	45.7	44.1
3-dimethylaminophthalimide	46.0	45.3	50.6	48.2	49.2
3-diphenylmethyl-2,4-pentanedione	48.4	51.6	50.5	52.1	55.9
3-ethoxy-4-hydroxybenzadehyde	38.2	39.4	40.5	39.4	40.3
3-ethylbenzoic acid	36.5	37.6	44.4	39.6	38.6
3-iodobenzoic acid	48.4	37.5	50.2	37.6	39.5
3-methoxy4nitrobenzoic acid	61.5	53.7	44.7	59.1	61.4
3-methyl-2,4,6-trinitrotoluene	57.1	57.0	41.8	57.1	57.0
3-methyl-3-nitro-4-N,N-dimethylamine	54.3	61.4	46.3	71.2	66.0
3-methylaminophthalimide	53.4	59.5	51.3	50.1	49.9
3-methylcholanthrene	68.5	67.6	60.8	68.9	68.7
3-nitroaniline	41.8	44.0	43.0	42.8	42.8
3-nitrophenol	36.7	40.8	41.8	41.1	41.2
3-phenyl-2-propynoic acid	42.1	40.9	47.8	41.8	38.8
3-pyridinecarboxamide	49.0	40.4	41.8	36.9	36.5
4,4-dimethyl-1,3-cyclohexanedione	36.3	25.7	35.7	29.0	29.4
4,5-tetramethylene-1,3-dithiolan-2-thione	46.4	35.3	34.8	48.0	49.7
4,5-tetramethylene-1,3-dithiole-2-thione	43.7	44.4	46.3	55.6	55.3
4-acetomidobenzaldehyde	44.1	44.1	42.9	40.3	39.9
4-amino-N-(2,5-dichlorophenyl) benzenesulfonamide	75.7	73.2	60.1	65.4	67.7

4-benzyloxyphenylacetic acid	57.8	57.7	46.8	60.9	58.9
4-chloroaniline	25.5	28.7	33.5	25.4	25.3
4-heptylbenzoic acid	53.9	53.8	58.1	52.9	52.2
4-hydroxy-4-nitroazobenzene	72.3	73.3	47.9	70.7	63.0
4-methoxy-benzenepropanoic acid	48.4	43.7	45.8	48.2	47.3
4-methylpyridine-N-oxide	37.8	30.9	33.3	27.4	26.2
4-nitro-4-(N,N-dimethyl)aminoazobenzene	72.9	60.3	47.4	71.2	64.5
4-nitrobenzoic acid	53.9	49.1	42.3	54.6	54.6
4-N,N-diethylaminoazobenzene	78.2	49.9	45.4	55.9	53.4
4-N,N-dimethylaminonitrosobenzene	54.4	48.7	39.1	33.8	33.9
4-octyl-benzoic acid	56.4	56.4	62.3	55.4	54.5
4-pentyl-benzoic acid	47.6	47.7	51.5	47.5	47.0
5-alpha-cholestane	61.3	61.4	61.0	61.3	61.3
5-aminoquinoline	43.7	39.5	50.2	39.5	36.5
5-chloro-2-hydroxy-benzophenone	45.0	45.9	42.7	46.4	47.6
5-chloro-7-iodo-8-hydroxy-quinoline	52.2	47.9	51.5	39.8	46.4
5-nitro-8hydroxyquinoline	49.3	56.6	51.7	61.1	60.1
5-phenyl-1,2-dithiole-3-thione	54.7	46.2	41.0	54.6	56.0
5-phenylvaleric acid	42.7	43.8	50.8	47.2	44.5
5-(trifluoromethyl)uracil	32.9	50.0	40.6	40.2	41.3
6-(chloro)uracil	67.8	40.1	39.6	38.8	40.0
7-bromo-5-chloro-8-hydroxyquinoline	49.5	46.7	49.1	39.8	44.8
8,16-pyranthenedione	114.4	114.3	103.0	114.2	114.3
8-aminoquinoline	33.2	39.3	45.4	39.5	36.5
8-butyl-9-methyladenine	63.2	63.5	53.3	63.2	61.1
8-ethyl-9-methyladenine	59.1	55.6	50.4	55.0	54.0
8-nitroquinoline	44.8	43.9	52.3	52.3	50.4
9,10-dimethylanthracene	51.2	46.8	52.4	42.9	44.6
9,9-bifluorenyl	71.3	88.9	78.6	89.5	90.6
9-hydroxy-1,4-anthracenedione	56.6	66.0	66.1	66.3	66.5
9-methyladenine	61.9	48.8	48.5	50.4	47.6
9-methylanthracene	42.2	42.3	50.7	42.1	41.0
Acetylsalicylic acid	43.6	43.7	43.7	51.1	52.3
adamantan-2-one	27.2	23.8	25.3	30.4	26.9
adamantylbromide	25.0	24.6	24.5	26.3	25.2
anthranthrene	78.0	77.8	72.1	77.8	78.1
a-tertbutyl-malononitrile	20.2	28.9	39.6	30.2	32.7
benzanilide	51.3	44.4	44.2	45.5	42.7
benzil	42.3	44.0	40.8	47.3	46.2
benzoapyrene	63.5	67.6	65.6	69.1	67.9
benzobfluorene	57.9	53.3	57.9	57.1	52.2
benzoyltrifluoracetone	24.0	36.1	40.5	40.7	41.3
bicyclo-2,2,2-octane	13.5	17.9	20.6	21.5	17.4
biphenyl	28.5	28.5	31.0	30.9	28.3
bis-2-chloroethylsulfide	21.4	20.3	40.8	24.0	28.3
cis-2-butenoic acid amide	26.3	31.6	40.8	26.1	26.5
cis-2-hexenoic acid amide	28.6	35.9	45.5	30.5	30.6
cis-2-pentenoic acid amide	31.3	33.4	43.7	28.2	28.5
coronene	83.0	88.1	84.3	83.5	85.9
cyanogendioxide	16.6	16.6	39.1	16.6	19.0
cyclopentadecanone	33.8	42.5	45.4	44.2	43.5
decafluorobiphenyl	25.7	37.5	28.5	40.8	50.6
dibenzo[fg,op]naphthacene	90.3	85.5	77.6	83.2	86.1
dibenzothiophene	38.3	39.6	49.3	47.1	43.8
diformylhydrazine	51.4	47.7	41.0	32.1	32.5
diphenylacetylene	34.2	33.7	36.8	38.2	34.2
diphenylcyclopropenone	55.1	45.9	42.6	46.6	42.2
disperseyellow	94.6	77.2	61.6	87.0	80.9
d,l-2,3-dimethoxy-2,3-diphenylbutane	41.9	51.8	44.9	45.2	51.5
dodecanamide	59.8	50.2	56.8	48.6	45.4

dodecanedioic acid	68.5	68.4	68.3	67.4	68.3
dodecanoic acid lauric acid	43.0	49.5	65.3	50.1	47.3
flurbiprofen	53.3	53.5	48.0	53.3	54.8
formic acid	5.7	29.6	37.2	20.5	20.1
heneicosanoic acid	66.6	66.4	86.2	66.6	66.8
hexachlorobenzene	44.2	27.5	37.7	22.6	34.4
hexadecenoic acid	55.5	59.4	63.9	59.9	57.6
hexakis(trifluoromethyl)tetracyclo [2.2.0.0(2,6).0(2,5)]hexane	7.8	46.3	45.8	45.1	47.3
hexamethylbenzene	30.6	29.7	30.8	22.6	25.3
homopiperonylic acid	50.4	51.9	53.6	52.4	50.4
LL-proline	64.6	63.6	60.4	64.5	63.4
malononitrile	21.0	23.4	39.5	26.8	27.0
mefenamic acid	59.2	62.1	47.0	57.0	58.7
methyl-butanedioic acid	48.6	48.6	48.5	49.1	49.6
methylcarbamate	22.5	31.7	40.7	27.2	28.0
methylmalonic acid	47.0	47.6	44.9	46.9	46.8
N-acetyl-1-naphthylamine	47.9	46.7	54.0	44.4	42.8
N-acetyl-glycine-N-methylamide	49.4	45.4	46.9	36.2	37.2
N-acetyl-L-isoleucineamide	74.2	54.0	50.3	43.8	46.9
naphthalene	22.4	22.5	39.8	25.6	22.1
N-benzyl-pivalophenoneimine	39.4	47.1	46.1	48.0	48.1
N-ethyl-N-methylUrea	42.5	30.8	40.3	27.7	29.1
Niflumic acid	61.3	67.1	45.9	64.5	64.7
N-methyl-N-nitromethanamine	19.0	20.3	32.8	26.9	28.0
N-methyltetradecanamide	49.5	55.5	68.6	54.1	50.1
N-methylthiourea	50.2	50.4	41.1	28.3	30.2
N-methylurea	57.5	50.2	42.4	32.5	34.8
N-nitromorpholine	19.9	27.5	32.3	37.7	37.3
N,N,N,N-tetramethyl-1,5-naphthalenediamine	39.2	46.2	47.2	38.5	42.7
N-[N-(N-[trifluoroacetyl]glycyl)glycyl]glycine methyl ester	60.0	59.5	48.1	57.9	56.9
n-octadecane	39.2	49.7	56.1	53.4	48.1
nonamide	44.2	43.4	55.0	38.7	37.6
N-phenyl-anthranilic acid	58.9	59.7	49.0	55.5	55.7
N-phenylbenzylamine	36.9	38.8	36.1	40.5	36.3
octanamide	42.0	40.5	52.2	35.8	35.1
oxalic acid monoamide	52.6	52.7	64.2	56.2	52.8
paracetamol	60.0	47.9	44.3	38.9	38.2
PCB126	57.8	39.6	48.3	37.2	47.6
PCB153	51.7	42.3	50.4	37.8	51.5
PCB174	55.1	44.4	54.5	37.8	55.4
PCB22	40.0	35.7	40.6	34.3	39.9
PCB70	47.2	37.9	44.9	35.6	43.7
PCDD26	56.5	56.6	59.0	60.3	63.0
PCDD27	57.5	61.6	60.9	59.9	68.6
PCDD65	68.2	72.4	67.9	63.3	78.7
PCDF2	40.0	40.2	51.6	43.4	41.6
pentacene	96.6	76.0	69.6	82.4	75.9
pentafluorophenol	14.4	28.9	27.9	26.9	29.7
pentamethoxycarbonylbenzene	73.3	73.4	71.3	73.2	73.5
pentamethylbenzene	26.2	27.0	29.4	21.8	23.1
perylene	72.7	67.5	65.4	67.2	68.0
phenazine	23.8	39.6	51.1	50.2	44.4
phenothiazine	49.5	51.7	54.4	63.5	58.5
phenylsalicylate	36.1	47.2	38.1	51.9	49.5
picene	83.2	76.1	69.8	79.3	76.4
proline,1-[N-(trifluoroacetyl)-1-leucyl] methyl ester	50.6	62.7	60.0	68.2	68.9
pterphenyl	56.8	42.8	38.7	48.5	45.5

ptoluidine	20.7	28.1	35.6	25.4	24.1
pyracene	46.5	46.5	56.3	48.0	46.6
resorcinol-di-benzoate	69.2	62.5	48.3	75.1	69.2
sesamol	32.0	35.6	46.0	37.0	33.1
succinimide	36.7	31.0	33.7	29.8	28.9
tetradecanamide	64.1	59.5	73.0	55.6	50.9
tetrahydro-4H-thiopyran-4-one	20.1	20.4	29.9	29.5	30.2
tetraphenylmethane	71.5	61.3	50.2	56.9	63.1
thiocamphor	25.3	27.8	30.8	29.7	30.9
trans-2-nonenoic acid amide	44.3	44.7	52.9	38.7	37.6
trans-2-octenoic acid amide	33.0	57.3	51.2	52.3	51.8
trans-3,5-dimethoxycinnamic acid	62.9	51.6	46.2	53.7	55.3
trans-3-phenyl-2-propen-1-ol	31.4	31.2	39.0	29.9	28.1
trichloro-1,4-benzoquinone	36.4	29.0	41.1	30.2	36.1
trifluoroacetamide	19.8	29.8	40.7	25.4	26.6
triphenylamine	41.8	45.5	41.9	48.2	49.3
triphenylene	65.2	56.7	60.2	57.1	57.0
Violanthrene-B	103.4	103.6	103.2	103.2	103.3

Test set:

	Expt.	Eq. 13	Eq. 14	Eq. 15	Eq. 16
1,3,5-trimethyl uracil	41.4	28.2	34.5	33.4	35.2
1,3-dimethyl-5-butyluracil	40.7	54.5	37.8	51.1	58.2
1,3-dimethyl-5-propyluracil	25.8	33.0	36.8	38.0	40.5
1-naphthylamine	33.0	36.8	44.3	34.4	31.8
2,2-dicyanopropionitrile	21.0	34.1	30.3	36.3	39.1
2,3 dimethylnaphthalene	30.6	30.2	37.3	28.6	27.6
2,4,6-trimethyl phenol	27.9	29.0	29.9	25.4	25.8
2,5-thiophenedicarboxylic acid	72.7	54.9	40.7	57.4	61.7
2-aminobenzoic acid	42.8	50.6	30.8	45.4	44.2
2-methoxybenzoic acid	43.7	38.2	33.3	42.1	41.9
3-benzalphthalide	57.7	52.4	45.7	57.7	53.3
3-tert-butylphenol	27.7	34.1	32.9	27.2	27.5
4-hexylbenzoic acid	50.5	51.1	39.9	50.2	49.7
4-phenylbutyric acid	39.0	40.2	33.5	44.4	41.7
5,7-dihydro-6H-dibenzo(a,c) cyclohepten-6-one	70.3	51.2	48.6	49.8	49.3
5-methyl-2-nitrobenzoic acid	51.6	60.4	32.6	55.6	57.7
9-butylanthracene	45.6	56.0	50.8	53.3	54.1
acetate-1-Naphthalenol	34.1	38.9	40.9	43.3	41.9
acridine	41.0	38.5	47.3	45.4	40.4
benzene	0.9	15.4	21.0	17.8	13.9
Butylparaben	42.2	46.9	38.3	46.3	45.2
d-3-bornanone	20.0	25.8	25.4	25.6	25.2
di-tert butylmethanol	16.3	28.9	30.7	21.7	23.5
fluorene	34.7	34.6	43.7	37.5	33.6
heptanamide	37.9	37.7	38.8	33.0	32.8
Methylparaben	42.2	39.6	36.3	37.3	37.6
N,N-tri-fluoroacetyl-valyl-alanine-ethyl ester	50.5	63.0	45.9	65.4	66.5
N-tert butylurea	59.8	45.3	32.3	31.3	32.6
PCB25	39.6	35.7	27.8	34.4	39.8
PCDF43	48.6	50.0	49.2	46.3	52.1
ppDDT	52.9	46.3	32.5	41.4	52.2
salicylic acid	38.5	48.9	35.8	43.7	42.4
tri-chloro-hydroquinone	40.4	50.7	30.4	32.3	38.1
tropolone	25.6	31.5	30.1	30.2	30.1
